# Taming interleukin‐12: Engineering of bispecific antibody‐based IL‐12 mimetics with biased agonism capacities

**DOI:** 10.1002/pro.70072

**Published:** 2025-02-21

**Authors:** Britta Lipinski, Laura Unmuth, Paul Arras, Ron Endruszeit, Stefan Becker, Jonathan Mathias Rödel, Jürgen Scheller, Silke Pudewell, Doreen M. Floss, Simon Krah, Julia Harwardt, Achim Doerner, Laura Helming, Chunxiao Xu, Andreas Menrad, Andreas Evers, Harald Kolmar, Desislava Elter, Lukas Pekar, Stefan Zielonka

**Affiliations:** ^1^ Biomolecular Immunotherapy, Institute for Organic Chemistry and Biochemistry Technical University of Darmstadt Darmstadt Germany; ^2^ Antibody Discovery and Protein Engineering Merck Healthcare KGaA Darmstadt Germany; ^3^ Institute of Biochemistry and Molecular Biology II Medical Faculty and University Hospital Düsseldorf, Heinrich‐Heine‐University Düsseldorf Düsseldorf Germany; ^4^ Research Unit Oncology EMD Serono Research Center Billerica Massachusetts USA; ^5^ Applied Biochemistry, Institute for Organic Chemistry and Biochemistry Technical University of Darmstadt Darmstadt Germany

**Keywords:** antibody engineering, bispecific antibody, cytokine mimetic, IL‐12, NK cell, single‐domain antibody, surrogate cytokine, T cell, VHH, yeast surface display

## Abstract

In this work, we have generated bispecific interleukin (IL)‐12 surrogate agonists based on camelid‐derived single‐domain antibodies (sdAbs) targeting the IL‐12 receptor (IL‐12R) subunits IL‐12Rβ1 and IL‐12Rβ2. Following immunization and antibody display‐based paratope isolation, respective sdAbs were combinatorially reformatted into a monovalent bispecific architecture by grafting resulting paratopes onto the hinge region of a heterodimeric Fc region. Functional characterization using NK‐92 cells enabled the identification of multiple different sdAb‐based bispecifics displaying divergent IL‐12R agonism capacities as analyzed by STAT4 phosphorylation. Further investigations by harnessing peripheral blood mononuclear cells (PBMCs) from healthy donors revealed attenuated pSTAT4 activation compared to recombinant human (rh) wild‐type IL‐12 regarding both natural killer (NK)‐cell and T‐cell activation but robust IL‐12R agonism on stimulated T cells. While several sdAb‐based IL‐12 mimetics were nearly inactive on NK cells as well as T cells obtained from PBMCs, they elicited significant STAT4 phosphorylation and interferon (IFN)‐γ release on stimulated T cells as well as an IL‐12‐like transcriptional signature. Furthermore, we demonstrate that the activity of receptor agonism of generated bispecific IL‐12 mimetics can also be biased towards stimulated T cells by changing the spatial orientation of the individual sdAbs within the molecular design architecture. Taken together, we present an alternative strategy to generate IL‐12‐like biologics with tailor‐made characteristics.

## 1 INTRODUCTION

Interleukin (IL)‐12 is a potent proinflammatory cytokine with substantial therapeutic potential (Cirella et al. 2022). IL‐12 was identified in the late 1980s by its ability to elicit IFN‐γ secretion in PBMCs (Kobayashi et al. 1989; Stern et al. 1990). From a structural perspective, IL‐12 is a heterodimeric cytokine consisting of the p35 α‐subunit and the p40 β‐subunit. The cognate receptor, termed IL‐12R, also comprises two subunits, IL‐12Rβ1 and IL‐12Rβ2. Of note, also other cytokines belonging to the IL‐12 cytokine family, such as IL‐23 or IL‐35, share one of the receptor subunits with IL‐12 (Hildenbrand et al. 2022). Primarily, the cellular sources for IL‐12 production are antigen presenting cells including dendritic cells, phagocytes and B cells upon sensing of pathogen‐associated molecular patterns (D'Andrea et al. 1992). IL‐12 mainly targets NK cells and T cells; however, it has been described that IL‐12R is also expressed on other immune cell subsets (Airoldi et al. 2000; Trinchieri 2003). Binding of IL‐12 to its receptor triggers the initiation of the JAK/STAT pathway, most importantly it facilitates STAT4‐mediated production of IFN‐γ (Yi et al. 2024). In addition, IL‐12 promotes polarization of CD4^+^ T cells into a T helper 1 (Th1) phenotype (Trinchieri 2003). The proinflammatory nature of IL‐12 renders this cytokine as attractive molecule for biomedical applications. Hence, IL‐12 has been investigated in clinical trials for cancer immunotherapy (Del Vecchio et al. 2007; Lasek et al. 2014). Unfortunately, IL‐12 administration into patients comes with the price of dose‐limiting toxicities, clearly hampering its clinical utility (Jia et al. 2022; Leonard et al. 1997). To avoid toxicities associated with systemic IL‐12 delivery, it might be beneficial to preserve IL‐12 functionalities especially on antigen‐experienced T cells within the tumor microenvironment, while sparing systemic T‐cell and NK‐cell activation. In this context it is worth mentioning that T cells upregulate IL‐12R expression upon activation via antigen stimulation through the T‐cell receptor (Glassman et al. 2021; Szabo et al. 1997).

In 2021, Garcia and co‐workers described an elegant approach for the engineering of IL‐12 based partial agonists by reducing the affinity of p40 for IL‐12Rβ1 (Glassman et al. 2021) which surface expression on T cells is significantly enhanced after stimulation. The resulting IL‐12 muteins were capable of inducing IFN‐γ secretion by CD8^+^ T cells with impaired NK‐cell activation. In a similar fashion, Feige and colleagues were able to induce a bias towards CD8^+^ T‐cell activation versus NK‐cell activation by lowering the affinity of p40 for IL‐12Rβ1 (Liebl et al. 2023). Besides, also other methods have been described that drag the proinflammatory function of IL‐12 to a specific cell subset in the tumor microenvironment (TME). In this regard, immunocytokines were constructed that deliver IL‐12 to the tumor site by fusing the cytokine to a TME targeting antibody paratope (Nadal et al. 2022; Pasche et al. 2012; Puca et al. 2020a; Puca et al. 2020b). In addition, Zou et al. constructed a low‐affinity version of IL‐12 that was fused to an anti‐PD‐1 antibody for cis‐delivery (Zou et al. 2024). PD‐1 is expressed at much higher levels on tumor infiltrating lymphocytes compared with T cells and NK cells in the periphery (Sharpe and Pauken 2018; Zou et al. 2024). Hence, the low affinity derivative of IL‐12 fused to a high affinity PD‐1 antibody preferentially activates tumor infiltrating PD1^+^ T cells, thus, eliciting robust antitumor activities in preclinical mouse models while exhibiting a beneficial toxicity profile (Zou et al. 2024). Of note, also other next‐generation engineering approaches of IL‐12 have been conducted, for instance, a masking approach that relies on a tumor protease cleavable linker (Lasek et al. 2014; Mansurov et al. 2022).

Herein, we present an alternative route for generating molecules displaying an IL‐12‐like function biased to pre‐activated T cells. Essentially, in this approach we exploit sdAb‐based bispecifics that mimic the function of IL‐12 by triggering IL‐12R agonism. Such molecules, referred to as cytokine mimetics or surrogate agonists have been successfully engineered in the past, as reviewed elsewhere (Pekar et al. 2024). In this respect, sdAb‐derived surrogate agonists have been constructed that mimic the function of IL‐2, type I interferons and IL‐18 (Harris et al. 2021; Lipinski et al. 2023a; Yen et al. 2022). Moreover, it was shown by the group of Christopher Garcia that also novel cytokine specificities can be engineered by targeting receptor heterodimers which do not exist naturally (Yen et al. 2022). In this present work, we immunized camelids with both (rh) IL‐12R subunits. By harnessing yeast surface display and phage display we were able to identify a set of IL‐12Rβ1 and IL‐12Rβ2 specific variable domains of heavy‐chain‐only antibodies (VHHs), respectively (Valldorf et al. 2021). Combinatorial reformatting into a strictly monovalent (1 + 1) bispecific antibody architecture and screening of hundreds of different bispecific combinations enabled the identification of IL‐12R agonists with different levels of agonism capacities. Intriguingly, several bispecific IL‐12 mimetics were almost inactive on NK and T cells obtained from PBMCs but triggered robust IL‐12R activation and IFN‐γ release on pre‐stimulated T cells with divergent potencies and efficacies. In addition, we present an alternative strategy to bias generated mimetics towards pre‐activated T cells by antibody engineering.

## 2 RESULTS

### 2.1 Isolation and generation of single‐domain antibodies specific to IL‐12Rβ1 and IL‐12Rβ2

A llama (*Lama glama*) and a huarizo (*Lama glama × Vicugna pacos*) were immunized four times with a cocktail (1:1) of recombinant human (rh) IL‐12Rβ1 and (rh) IL‐12Rβ2. For the isolation of specific VHHs against both receptor subunits yeast surface display (YSD) libraries were generated for each animal and sorted individually. Regarding YSD, by applying a two‐dimensional sorting strategy to co‐select for full‐length VHH display in addition to the binding functionality, we observed a strong enrichment of (rh) IL‐12Rβ1 binding populations within two to three rounds of fluorescence activated cell sorting (FACS) using an antigen concentration of 1 μM (Figure S1a, Supporting Information) (Roth et al. 2020). In the instance of IL‐12Rβ2, however, the camelids were immunized with an Fc‐tagged antigen. Unfortunately, this resulted primarily in the enrichment of Fc‐binding VHH variants for the library constructed based on the immunized huarizo. This became evident when we used an irrelevant antigen‐Fc fusion protein for staining (data not shown). Also, in the case of the library derived from the immunized llama we co‐enriched for Fc‐binding populations in addition to IL‐12Rβ2 variants within two rounds of FACS. Consequently, we conducted a negative sorting round by employing an irrelevant antigen‐Fc fusion and selected for full‐length display only (Figure S1b), enabling a preferential enrichment of IL‐12Rβ2‐specific VHH domains. To further identify IL‐12Rβ2‐targeting sdAbs, we also constructed a phage display library based on the PBMC repertoire of immunized camelids. This was achieved by applying a Golden Gate cloning based approach, as previously described by our group (Bauer et al. 2023; Sellmann et al. 2020). Three rounds of panning were conducted, using plate‐immobilized (rh) IL‐12Rβ2. Binding assessment by ELISA revealed 15 positive hits which were sent out for sequencing (Figure S1c). By applying a clustering strategy based on the diversity within CDR3 after sequencing of clones obtained from both display platforms, we nominated 39 VHHs targeting IL‐12Rβ1 and 50 targeting IL‐12Rβ2 for reformatting and expression (Figure S1d).

For antibody production we exploited the strand‐exchange engineered domain (SEED) technology resulting in preferential heavy chain heterodimerization owing to β‐strand exchanges of constant regions CH3 of IgG and IgA (Davis et al. 2010). In this regard, respective IL‐12Rβ1 addressing VHHs were grafted onto the hinge region of the Fc‐effector silenced AG chain of the SEEDbody, whereas IL‐12Rβ2‐specific sdAbs were fused to the *N*‐terminus of the Fc‐effector silenced GA chain. Monospecific constructs (1 + 0), where the VHH‐AG or VHH‐GA fusion was paired with a paratope‐less respective GA or AG chain were expressed in Expi293™ cells. After protein A purification, specific binding of the monospecific constructs to the (rh) ECDs of IL‐12Rβ1 and IL‐12Rβ2 was assessed by biolayer interferometry (BLI). To this end, either (rh) His‐tagged IL‐12Rβ1 or IL‐12Rβ2 was loaded onto the biosensors and monospecific SEEDbodies were utilized at a single concentration of 100 nM. Ultimately, 29 clones displayed binding to (rh) IL‐12Rβ1 and 12 clones for IL‐12Rβ2, respectively (Figure S1e). Importantly, none of the IL‐12Rβ2‐specific sdAbs showed binding to human Fc (data not shown).

### 2.2 Identification of bispecific IL‐12 receptor agonists triggering STAT4 activation

**FIGURE 1:**
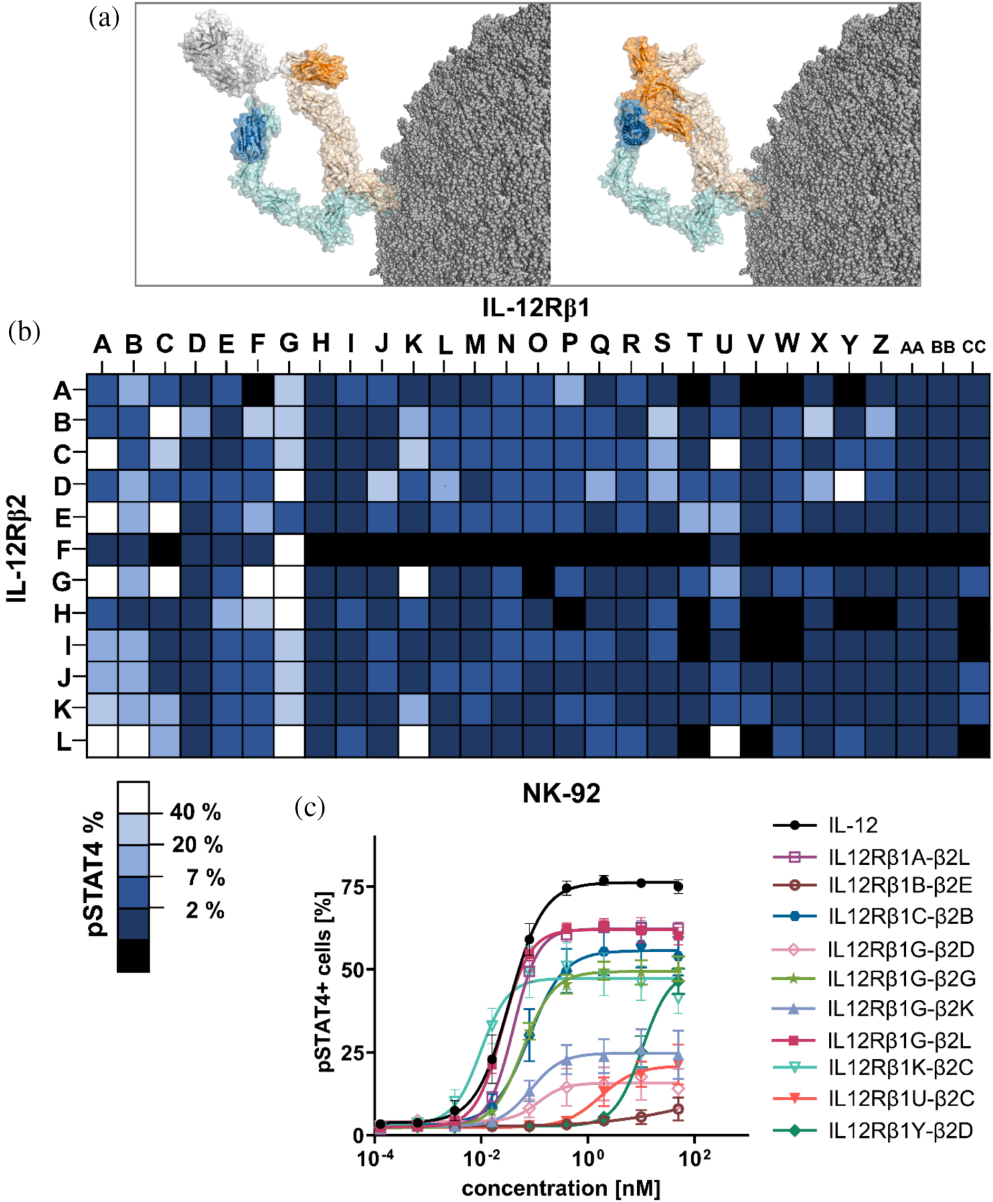
Combinatorial reformatting of sdAbs targeting IL‐12Rβ1 and IL‐12Rβ2 into a bispecific antibody architecture (1 + 1) enables screening of IL‐12R surrogate agonists. (a) Left: bispecific single‐domain antibody binding to the heterodimeric IL‐12R by engaging IL‐12Rβ1 (light orange) and IL‐12Rβ2 (light blue) and eliciting downstream signaling. Right: heterodimeric IL‐12 initiates a complex with its own receptor through the subunits p35 (IL12A, blue) and p40 (IL12B, orange). Structural visualization was generated with PyMOL software version 2.3.0, based on PDB entry 8ODZ and structural modeling as described in section 4. (b) Heatmap of NK‐92 STAT4 phosphorylation capacity of IL‐12R agonists by combinatorial reformatting. Molecules failing expression are displayed in black, percentage of pSTAT4^+^ cells for active agonists are depicted in dark blue (<2%) to visualize functionally inactive bsAbs, while lighter blue shades (<7%, <20%, <40%) are given for moderately active surrogate agonists. The highest agonism is shown for constructs in white (>40%). Data from two independent experiments are depicted. (c) NK‐92 cells were incubated with IL‐12 and surrogate agonists in varying concentrations for 45 min. Prior to pSTAT4 staining, cells were fixed and permeabilized. Mean values ± SEM of three independent experiments are shown.

In a first attempt to characterize the sdAbs regarding their capability to mimic the function of IL‐12, the selected 29 sdAbs specific to IL‐12Rβ1 (referred to as IL12Rβ1A‐IL12Rβ1CC) were reformatted with each of the 12 sdAbs specific to IL‐12Rβ2 (named IL12Rβ2A‐IL12Rβ2L) in a combinatorial manner resulting in 348 (monovalent for each receptor subunit, 1 + 1) bispecific antibodies (bsAbs) (Figure 1a). Expression in 5 mL ExpiCHO‐S™ led to the exclusion of 42 constructs due to insufficient production yields (shown in black). For all others, we exploited the immortalized NK cell line NK‐92 to assess STAT4 phosphorylation after 45 min of incubation with the bsAbs. To this end, three concentrations (250, 12.5, and 0.625 nM) of the IL‐12R‐targeting bsAbs were used as well as (rh) IL‐12 as positive control. The highest percentage of STAT4‐activated cells for each construct after assay repetition is illustrated in Figure 1b, clearly demonstrating a wide range of agonism triggered by the different IL‐12R‐specific bsAbs. In this respect, among all 306 analyzed bispecifics, 161 molecules (53%) triggered activation of the STAT4 pathway (>2% pSTAT4‐positive NK‐92 cells). While 20% (57 constructs) displayed more than 7% STAT4 activation, 19 mimetics were able to agonize IL‐12R on NK‐92 cells to a higher extent, showing more than 40% STAT4 phosphorylation. Intriguingly, one particular IL‐12Rβ1‐targeting sdAb (IL12Rβ1G) elicited IL‐12R agonism when recombined with almost all IL‐12Rβ2‐specific VHHs. Competition experiments with (rh) IL‐12 for binding to (rh) IL‐12Rβ1 revealed non‐competitive binding, indicating that this specific sdAb paratope targets an epitope on the IL‐12Rβ1 different from (rh) IL‐12 that might be privileged in triggering receptor activation (Figure S2).

Furthermore, we set out to confirm IL‐12 agonism in a dose‐dependent manner for a subset of generated surrogate agonists. For this, NK‐92 cells were incubated with 49 bsAbs in varying concentrations (Figure S3). This unveiled dose‐dependent STAT4 phosphorylation of NK‐92 cells for almost all bsAbs tested. Substantial differences in potencies (EC_50_ of STAT4 phosphorylation) as well as efficacies (*E*_max_ STAT4 activation) were observed in this screening experiment (*N* = 1–2 for each molecule). Moreover, for some of the bispecifics, we noticed a hooking effect of STAT4 activation at high concentrations (which might be physiologically irrelevant). This bell‐shaped activity curve arises from the saturation of both receptor subunits, leading to inefficient engagement of the IL‐12 receptor. The phenomenon of inadequate receptor complex formation at elevated concentrations has been frequently documented for bispecific agonists (Claus et al. 2023; Lipinski et al. 2023a; Zhang et al. 2024).

Next, we selected 10 VHH‐based mimetics based on their differential IL‐12‐like profiles and favorable biophysical attributes (expression yields and target monomer peaks in analytical size exclusion chromatography post protein A purification; Table 1) to characterize their agonistic potential in more detail. The 10 different IL‐12 mimetics were again tested for their capability to trigger STAT4 phosphorylation on NK‐92 cells in three independent experiments, confirming strong differences in potencies and efficacies of agonism (Figure 1c and Tables 1, and S1). (rh) IL‐12 elicited quite potent STAT4 phosphorylation (EC_50_ = 0.03 nM, *E*_max_ of STAT4‐positive NK cells = 76%). Potencies (EC_50_ of pSTAT4) for generated IL‐12 mimetics ranged between 0.01 nM (IL12Rβ1K‐β2C) and 10.5 nM (IL12Rβ1Y‐β2D). BsAb IL12Rβ1B‐β2E elicited only negligible STAT4 activation at high concentrations.

**TABLE 1:** Biophysical, biochemical, and functional properties of the generated bispecific IL‐12 surrogate agonists.

Samples	Final yield (mg/L)	SEC purity after protein A (%)	Mean absolute pSTAT4 activation of NK‐92 cells (%)	EC_50_ pSTAT4 activation of NK‐92 cells (nM)
IL‐12			76	0.03
IL12Rβ1A‐β2L	108	100.0	62	0.04
IL12Rβ1B‐β2E	50	86.2	15	n.a.
IL12Rβ1C‐β2B	81	97.3	56	0.08
IL12Rβ1G‐β2D	66	95.7	16	0.12
IL12Rβ1G‐β2G	40	91.1	49	0.06
IL12Rβ1G‐β2K	102	98.6	25	0.08
IL12Rβ1G‐β2L	89	98.7	62	0.03
IL12Rβ1K‐β2C	104	97.8	47	0.01
IL12Rβ1U‐β2C	118	93.6	21	1.7
IL12Rβ1Y‐β2D	74	100.0	50	10.5

The identical IL‐12Rβ1‐targeting sdAb paratope IL12Rβ1G displayed significant differences in terms of agonism capacities when reformatted with distinct IL‐12Rβ2‐specific sdAbs (Figure 1c; IL12Rβ1G‐β2D vs. IL12Rβ1G‐β2G vs. IL12Rβ1G‐β2K vs. IL12Rβ1G‐β2L). While IL12Rβ1G‐β2L as well as IL12Rβ1G‐β2G robustly triggered IL‐12R agonism (EC_50_ = 0.03 nM, *E*_max_ of STAT4‐positive NK‐92 cells = 62% and EC_50_ = 0.06 nM, *E*_max_ = 49%, respectively), IL12Rβ1G‐β2D and IL12Rβ1G‐β2K were only moderate agonizing NK‐92 cells (EC_50_ = 0.12 nM, *E*_max_ = 16% and EC_50_ = 0.08 nM, *E*_max_ = 25%, respectively). Of note, we did not observe a clear correlation between agonism potential and affinities of incorporated VHHs. For example, IL12Rβ2L as well as IL12Rβ2G which elicited robust STAT4 activation when reformatted as bispecific (1 + 1) with IL12Rβ1G (Figure 1c) were significantly different in their binding affinities against (rh) IL‐12Rβ2 (Figure S4; IL12Rβ2L: KD = 140 pM vs. IL12Rβ2G: KD = 59.5 nM). Additionally, also both IL‐12Rβ2‐adressing VHHs that triggered only moderate agonism when recombined with IL12Rβ1G were significantly different in their binding affinities (IL12Rβ2D: KD = 115 nM vs. IL12Rβ2K: KD = 1.9 nM).

With respect to epitope targeting, it is worth mentioning that IL12β2D and IL12Rβ2K share either an overlapping epitope on IL‐12Rβ2 or address an epitope in close proximity, as revealed by binning experiments using BLI. Here, (rh) IL‐12Rβ2 was immobilized on the sensor tips, followed by successive associations of the two cytokine mimetics (Figure S2). While in one orientation (IL12Rβ2D followed by IL12Rβ2K) consecutive association was observed, IL12Rβ2D was prevented from binding to (rh) IL‐12Rβ2 when a prior association step was performed using IL12Rβ2K. In contrast to this, IL12Rβ2L, which elicited relatively strong agonism when recombined with IL12Rβ1G, targeted a different epitope compared with IL12Rβ2D, IL12Rβ2K as well as IL12Rβ2G. While addressing a different epitope on IL‐12Rβ2 than IL12Rβ2D, IL12Rβ2G, however, shared either an overlapping epitope with IL12Rβ2K or an epitope in close vicinity (Figure S2). Consequently, it is tempting to speculate that the fine epitope that is being addressed has major ramifications in terms of IL‐12R agonism capacities whereas affinities (within a certain range) seem to be less important. This was also evident for sdAbs targeting IL‐12Rβ1, where profound differences in affinities were observed for bsAbs triggering robust receptor activation (KD_IL12Rβ1_ ranging from the pM range to the triple digit nM range; Figure S4a). Also, for identical IL‐12Rβ2‐specific sdAbs we noticed substantial differences in terms of both, agonism potencies and efficacies when reformatted with IL‐12Rβ1‐targeting VHHs that address different epitopes on IL‐12Rβ1 (IL12Rβ1K‐β2C vs. IL12Rβ1U‐β2C and IL12Rβ1G‐β2D vs. IL12Rβ1Y‐β2D; Figures 1c and S2 and Table 1). This is clearly highlighting that within a given bispecific antibody architecture the epitopes that are being addressed on individual receptor subunits are of utmost importance in terms of inducing different IL‐12 heterodimer geometries eventually resulting in a broad diversity regarding agonism capacities.

### 2.3 VHH‐derived IL‐12 mimetics harbor distinct biases in preferentially agonizing pre‐activated T cells over non‐stimulated NK cells and T cells

Since it is generally appreciated that dose‐limiting toxicities associated with IL‐12 treatment are a direct consequence of immune cell activation in the circulation and periphery (Jia et al. 2022), we investigated whether distinct generated surrogate agonists display a bias compared to IL‐12 with reduced non‐stimulated T‐cell and NK‐cell activation while maintaining robust activation of stimulated T cells. For this, we considered IL‐12 mimetics IL12Rβ1G‐β2K, IL12Rβ1G‐β2L, IL2Rβ1U‐β2C, and IL‐12Rβ1Y‐β2D based on their divergent profiles in inducing STAT4 phosphorylation in NK‐92 cells (Figure 1c and Table 1). While IL12Rβ1G‐β2L elicited strong NK‐92 cell activation, IL12Rβ1G‐β2K and IL12Rβ1U‐β2C were only moderately triggering STAT4 phosphorylation with differences in potencies between the two variants. Finally, IL12Rβ1Y‐β2D robustly facilitated agonism of IL‐12R on NK‐92 cells (Table 1; EC_50_ = 10.5 nM), yet, with substantially attenuated potencies (attenuation: 350‐fold) compared with (rh) IL‐12 (Figure 1c and Table 1; EC_50_ = 0.03 nM).

**FIGURE 2:**
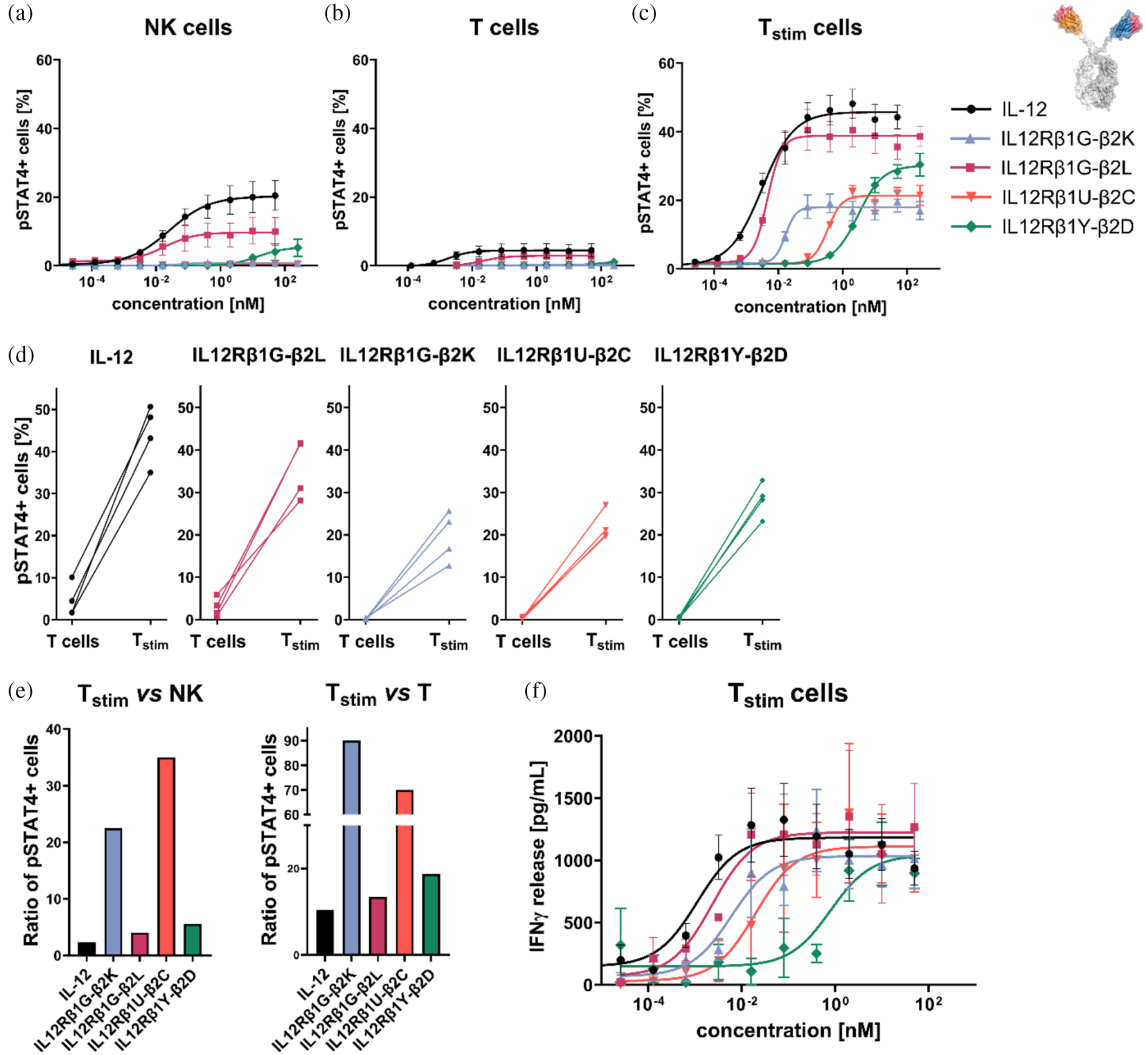
IL‐12 mimicking sdAb‐based surrogate agonists preferentially activate stimulated T cells with reduced activity on NK and T cells. (a–c) Intracellular staining of pSTAT4‐positive cells after 45 min incubation with IL‐12 or IL‐12 surrogate agonists (IL12Rβ1G‐β2K, IL12Rβ1G‐β2L, IL12Rβ1U‐β2C, or IL12Rβ1Y‐β2D). Graphs display mean values ± SEM of three (NK cells) to four independent experiments. (d) Absolute STAT4 phosphorylation in T cells displayed for each donor depending on their stimulation state. (e) Ratio of maximal pSTAT4 signaling of stimulated T cells vs. NK or vs. T cells. (f) IFN‐γ levels measured by HTRF in supernatant of stimulated T cells after 24 h stimulation with IL‐12 or IL‐12 surrogate agonists (IL12Rβ1G‐β2K, IL12Rβ1G‐β2l, IL12Rβ1U‐β2C, or IL12Rβ1Y‐β2D) in a dose–response curve. Mean values ± SEM of four independent experiments are shown.

We specifically scrutinized the agonism profiles of engineered VHH‐derived IL‐12 mimetics in comparison to (rh) IL‐12 on NK cells and T cells within the PBMC repertoires from healthy donors. From the same donors, we also stimulated T cells for 7 days as described in the methods section. In accordance with published data (Glassman et al. 2021) this induced the expression of the IL‐12Rβ1 subunit as well as surface expression of the activation and exhaustion markers PD‐1, TIM‐3, LAG‐3, and CTLA‐4 (Figure S5). In this regard, (rh) IL‐12 quite potently activated NK cells derived from PBMCs (Figures 2a and S6 and Table 2; EC_50_ = 0.03 nM, *E*_max_ = 20.2%) whereas it only induced STAT4 phosphorylation in a minor fraction of 4.4% of total T cells (Figures 2b and S6 and Table 2; EC_50_ = 0.002 nM). This is in line with higher expression profiles of the IL‐12Rβ1 subunit we observed on PBMC‐derived NK cells compared with T cells (Figure S5). In contrast to non‐stimulated T cells, pre‐activated T cells were strongly activated (Figures 2c and S6 and Table 2; EC_50_ = 0.003 nM, *E*_max_ = 46%) which was evident on all donors tested (Figure 2d). Based on the ratio of maximum STAT4 phosphorylation of stimulated T cells vs. non‐stimulated T cells or vs. NK cells, respectively, we were able to calculate a score for preferential stimulated T‐cell activation in a quantitative manner (Figure 2e; (rh) IL‐12: ratio T_stim_ vs. NK cells = 2.3 and ratio T_stim_ vs. T cells = 10.5).

**TABLE 2:** Agonistic activity of the herein generated four leading IL‐12 mimetics and engineered format.

Samples	NK cells		T cells		T_stim_ cells		T_stim_ cells	
	Absolute pSTAT4+ (%)	EC50 pSTAT4+ (nM)	Absolute pSTAT4+ (%)	EC50 pSTAT4+ (nM)	Absolute pSTAT4+ (%)	EC50 pSTAT4+ (nM)	IFN‐γ release (pg/mL)	EC50 IFN‐γ release (nM)
IL‐12	20.2	0.03	4.4	0.002	46	0.003	1221	0.001
IL12Rβ1G‐β2K	0.8	0.06	0.2	0.39	18	0.02	1034	0.01
IL12Rβ1G‐β2L	9.7	0.02	2.9	0.02	39	0.005	1283	0.003
IL12Rβ1U‐β2C	0.6	2.02	0.3	2.4	21	0.34	1110	0.02
IL12Rβ1Y‐β2D	5.4	14.8	1.6	n.a.	30	3.03	1037	0.68
aDIG_IL12Rβ1G‐β2L	3.8	0.11	0.8	0.14	27	0.07	1025	0.01

In comparison to (rh) IL‐12, the most potent VHH‐derived bispecific IL‐12 mimetic, IL12Rβ1G‐β2L triggered maximum STAT4 phosphorylation of non‐stimulated T cells and NK cells to a lesser extent, while activating stimulated T cells in a similar manner to the wild‐type cytokine (EC_50_ NK cells = 0.02 nM, *E*_max_ NK cells = 9.7%, EC_50_ T cells = 0.02, *E*_max_ T cells = 2.9%, EC_50_ T_stim_ = 0.005 nM, *E*_max_ T_stim_ = 39%). Hence, IL12Rβ1G‐β2L displayed a moderate bias for stimulated T‐cell support with reduced activity on NK cells or rested T cells (IL12Rβ1G‐β2L: ratio T_stim_ vs. NK cells = 4.0 and ratio T_stim_ vs. T cells = 13.4). Consistent with NK‐92 pSTAT4 profiles, surrogate agonist IL12Rβ1Y‐β2D was substantially attenuated in terms of potencies of NK‐cell, rested T‐cell and stimulated T‐cell activation (EC_50_ NK cells = 14.8 nM [493.3‐fold attenuation], EC_50_ T cells = n.a., EC_50_ T_stim_ = 3.03 nM [>1000‐fold]). Moreover, in the concentration range tested, this particular variant showed also reduced overall STAT4 phosphorylation (*E*_max_ NK cells = 5.4%, *E*_max_ T cells = 1.6%, *E*_max_ T_stim_ = 30%). Essentially, this resulted in a more pronounced bias for stimulated T‐cell activation (IL12Rβ1Y‐β2D: ratio T_stim_ vs. NK cells = 5.6 and ratio T_stim_ vs. T cells = 18.8). Strikingly, this bias was even much more prominent for IL‐12 mimetics IL12Rβ1G‐β2K (IL12Rβ1G‐β2K: ratio T_stim_ vs. NK cells = 22.5 and ratio T_stim_ vs. T cells = 90) and IL12Rβ1U‐β2C (IL12Rβ1U‐β2C: ratio T_stim_ vs. NK cells = 35 and ratio T_stim_ vs. T cells = 70). Both bsAbs were almost incapable of facilitating STAT4 phosphorylation in NK cells and rested T cells, while moderately agonizing IL‐12R on stimulated T cells: (IL12Rβ1G‐β2K: *E*_max_ NK cells = 0.8%, *E*_max_ T cells = 0.2%, *E*_max_ T_stim_ cells = 18%; IL12Rβ1U‐β2C: *E*_max_ NK cells = 0.6%, *E*_max_ T cells = 0.3%, *E*_max_ T_stim_ = 21%).

Of note, while differences were obtained for maximum STAT4 phosphorylation in distinct cell populations, all sdAb‐derived IL‐12 mimetics were capable of inducing maximum IFN‐γ release from pre‐activated T cells in a comparable manner to (rh) IL‐12 (Figure 2f and Table 2). In alignment with potencies in terms of STAT4 phosphorylation, also potencies for induction of IFN‐γ production (EC50_IFN‐γ_) were different for the four surrogate agonists. In this respect, IL12Rβ1G‐β2L behaved rather similar to (rh) IL‐12 in inducing IFN‐γ expression (IL12Rβ1G‐β2L: EC50_IFN‐γ_ = 0.003 nM vs. (rh) IL‐12: EC50_IFN‐γ_ = 0.001 nM). IL12Rβ1G‐β2K and IL12Rβ1U‐β2C were less potent in comparison with (rh) IL‐12 by a factor of 10 and 20, respectively (IL12Rβ1G‐β2K: EC50_IFN‐γ_ = 0.01 nM and IL12Rβ1U‐β2C: EC50_IFN‐γ_ = 0.02 nM). In stark contrast, IL12Rβ1Y‐β2D was substantially attenuated with reduced potencies of 680‐fold (IL12Rβ1Y‐β2D: EC50_IFN‐γ_ = 0.68 nM). Taken together, these findings are supporting the notion that VHH‐based IL‐12 mimetics can readily be created which display unique characteristics regarding both, signaling strength for agonism and cell population bias.

### 2.4 Antibody format engineering enables augmented bias towards stimulated T cells

**FIGURE 3:**
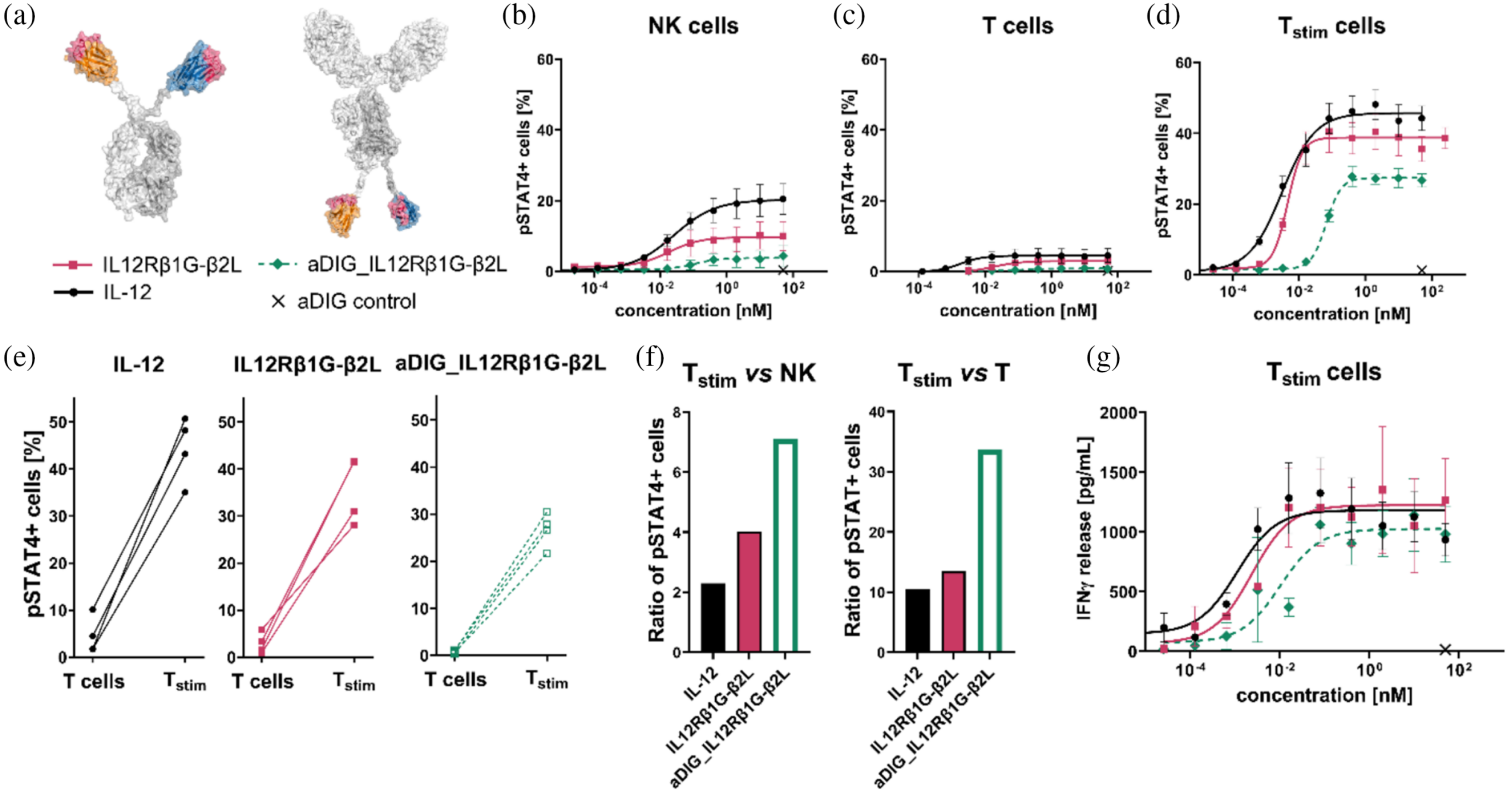
Antibody engineering enables generation of IL‐12 mimetic with pronounced bias for activated T cells. (a) Two different bsAb architectures incorporating paratopes IL12Rβ1G and IL12Rβ2L were functionally compared. For the *C*‐terminal IgG‐VHH format, VHHs were grafted to the *C*‐terminus of the heavy chain of an isotype control antibody (aDIG) by incorporating a 15 amino acid linker (3xGly_4_Ser). (b–d) Intracellular staining of pSTAT4^+^ cells after 45 min incubation with IL‐12 or IL‐12 surrogate agonists at different concentrations (IL12Rβ1G‐β2L and aDIG_IL12Rβ1G‐β2L). Graphs display mean values ± SEM of three (NK cells) to four independent experiments. (e) Absolute pSTAT4 signaling in T cells displayed for each donor depending on their stimulation state. (f) Ratio of maximal pSTAT signaling of pre‐activated T cells vs. NK or vs. T cells. (f) IFN‐γ levels measured by HTRF in supernatant of stimulated T cells after 24 h stimulation with IL‐12 or IL‐12 surrogate agonists (IL12Rβ1G‐β2L and aDIG_IL12Rβ1G‐β2L) at varying concentrations. Mean values ± SEM of four independent experiments are given.

In addition to profiling different generated sdAb‐based bsAbs, we also aimed at implementing a biased agonism by modulating the spatial orientation of individual VHH paratopes within the antibody architecture. In particular, we were interested in fusing the sdAbs of a given IL‐12 mimetic to the *C*‐terminus of an IgG, which might enable preferential delivery of the mimetic to a cognate cell population such as tumor cells or immune cells within the tumor microenvironment. To analyze a potential bias in an isolated manner, we fused the VHHs of IL12Rβ1G‐β2L to the *C*‐terminus of the heavy chain of a typical isotype control antibody in which the Fab arms targeted digoxygenin (aDIG). To this end, IL12Rβ1G was fused to the *C*‐terminus of one side of the heterodimer heavy chain, while IL12Rβ2L was grafted to the *C*‐terminus of the other heavy chain (Figure 3a). Between the Fc part and each individual VHH we incorporated a 15 amino acid linker (3xGly_4_Ser).

This molecule targeted both IL‐12R subunits with similar affinities as the parental IL‐12 mimetic (Figure S7; aDIG_IL12Rβ1G‐β2L: KD_IL‐12Rβ1_ = 1.3 nM, KD_IL‐12Rβ2_ = 0.4 nM vs. IL12Rβ1G‐β2L: KD_IL‐12Rβ1_ = 1.5 nM, KD_IL‐12Rβ2_ = 0.2 nM). However, we observed subtle differences in the maximum interference pattern shifts (BLI) against both receptor subunits. While aDIG_IL12Rβ1G‐β2L showed slightly increased binding capacities to (rh) IL‐12Rβ1 in comparison to the parental *N*‐terminal counterpart IL12Rβ1G‐β2L, we witnessed a trend towards reduced maximum interference pattern shifts against (rh) IL‐12Rβ2 in three independent experiments, suggesting a higher IL12Rβ1‐dependancy for aDIG_IL12Rβ1G‐β2L (Figure S7).

Regarding STAT4 phosphorylation of different cell populations, aDIG_IL12Rβ1G‐β2L displayed a more pronounced bias for preferential agonism of stimulated T cells (Figure 3f; aDIG_IL12Rβ1G‐β2L: ratio T_stim_ vs. NK cells = 7.1 and ratio T_stim_ vs. T cells = 33.8; IL12Rβ1G‐β2L: ratio T_stim_ vs. NK cells = 4.0 and ratio T_stim_ vs. T cells = 13.4). This was characterized by reduced potencies and efficacies primarily on NK and rested T cells as well as a milder attenuation on stimulated T cells for all donors tested (Figures 3b–f and S8 and Table 2). Also, with respect to the induction of IFN‐γ release from pre‐activated T cells only a mild attenuation in comparison to the parental *N*‐terminal SEED‐based molecule, IL12Rβ1G‐β2L, was observed (Figure 3g and Table 2; aDIG_IL‐12Rβ1G‐β2L: EC50_IFN‐γ_ = 0.01 nM, *E*_max_ = 1025 pg/mL). This provides further evidence that the spatial orientation of individual sdAbs within the overall antibody design architecture provides another angle for the optimization of IL‐12 agonism on specific cell populations.

### 2.5 mRNA sequencing reveals similar but distinct transcriptional signatures in stimulated T cells treated with the different IL‐12 mimetics or IL‐12

Finally, we performed mRNA sequencing of stimulated T cells treated with one of the four leading IL‐12 mimetics (IL12Rβ1G‐β2K, IL12Rβ1G‐β2L, IL12Rβ1U‐β2C, and IL12Rβ1Y‐β2D), as well as for the IL‐12 mimetic in the IgG‐VHH format (aDIG_IL12Rβ1G‐β2L) to investigate whether the transcriptional signatures induced by the compounds were similar to (rh) IL‐12 treatment. For this, isolated T cells of three independent donors were pre‐activated in accordance with preceding experiments and subsequently stimulated for 24 h with (rh) IL‐12 or the surrogate agonists. Principal components analysis (PCA) revealed the similarity among all samples of each donor with a tendency that the surrogate agonists had at least a comparable distance as (rh) IL‐12 on the PC1 axis to the untreated control (Figure S9a).

Overall, we observed upregulation of gene expression of 261 differentially expressed genes (DEGs) for (rh) IL‐12 (*p*_adj_ <0.05) (Figure S9b). While total upregulation of DEGs induced by the surrogate agonists were divergent (ranging from 209 DEGs for IL12Rβ1G‐β2K to 452 DEGs for IL12Rβ1G‐β2L), the total number of upregulated genes shared with (rh) IL‐12 matched fairly well with maximal STAT4 phosphorylation in stimulated T cells induced by the surrogate agonists. In this regard, IL12Rβ1G‐β2L, its *C*‐terminal counterpart aDIG_IL12Rβ1G‐β2L as well as IL12Rβ1Y‐β2D which all induced robust STAT4 activation shared 155 DEGs, 138 DEGs, and 156 DEGs with (rh) IL‐12, respectively. IL12Rβ1U‐β2C as well as IL12Rβ1G‐β2K which were inferior in eliciting maximal STAT4 activation only shared 127 as well as 103 upregulated DEGs with (rh) IL‐12.

**FIGURE 4:**
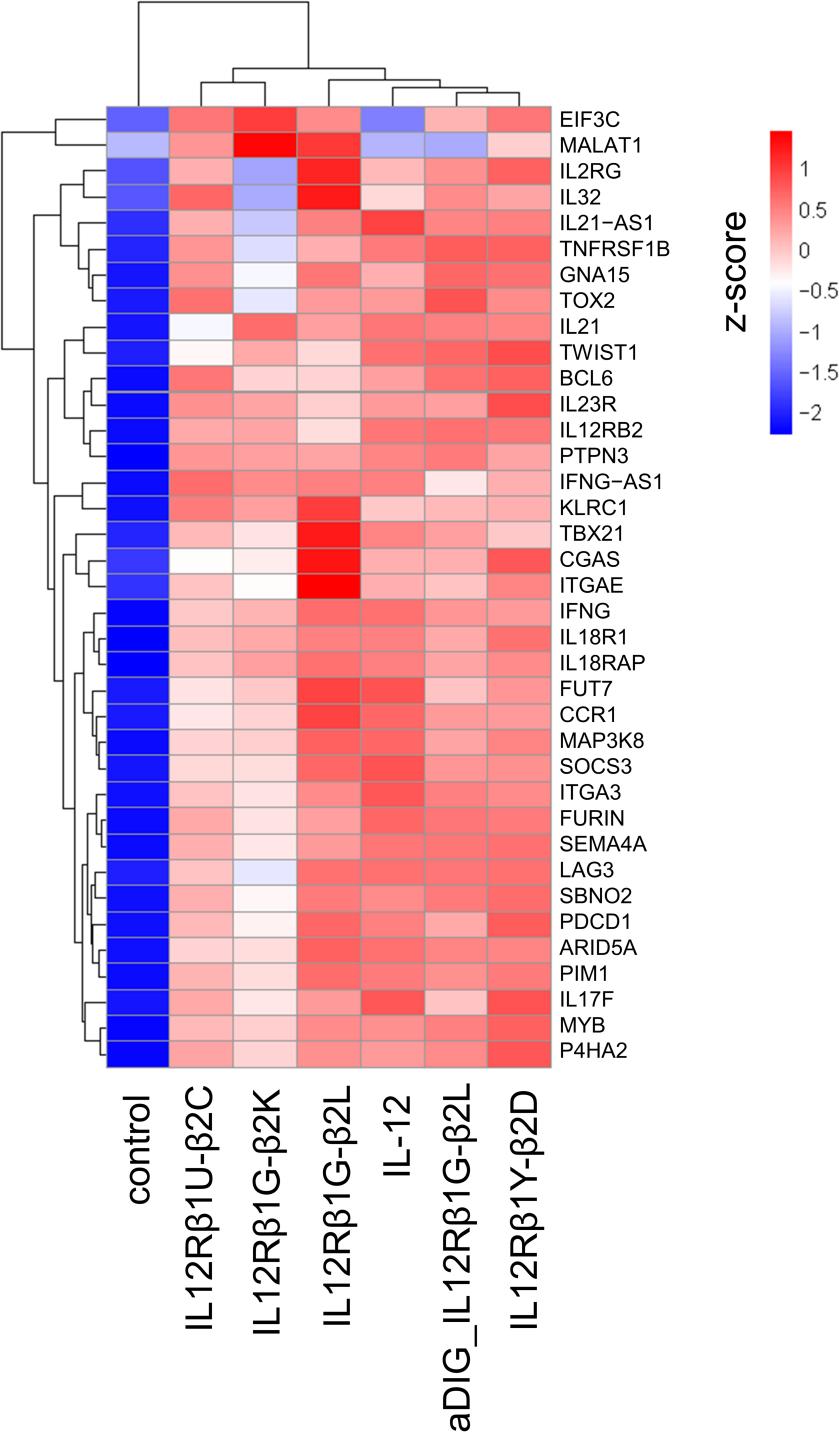
Hierarchical clustering of target DEGs by IL‐12 and antibody based surrogate agonists. Gene expression analysis in stimulated T cells from three donors treated with IL‐12 or surrogate agonists for 24 h. (a) Heatmap is generated based on FPKM values of differentially expressed genes with mainstream hierarchical clustering and normalized rows via z‐score (Gene expression value in sample of interest) − (Mean expression across all samples)/Standard Deviation). Genes are arrayed by row and agonists by column. IL‐12‐related genes that were aligned to the molecular signature database MSigDB (Liberzon et al. 2015; Subramanian et al. 2005) and significantly altered (*p*_adj_ <0.05) by IL‐12 or surrogate agonist treatments are displayed.

We further analyzed specific target genes known or supposed to be upregulated in IL‐12‐dependent T‐cell activation (Figure 4). In general, the transcriptional signature in stimulated T cells triggered by the different VHH‐derived surrogate agonists were rather similar to the gene expression profile elicited by (rh) IL‐12. For instance, we found a strong consensus of *IFNG*, *IL18R1*, *IL18RAP*, and *MAP3K8* that were significantly increased in all samples tested. In the displayed hierarchical cluster heatmap IL12Rβ1G‐β2K and IL12Rβ1U‐β2C grouped together since their facilitated transcriptional signatures were characterized by moderate upregulation of IL‐12‐related targets. Of note, also for those two mimetics that behaved similar in terms of STAT4 phosphorylation and IFN‐γ release we witnessed differential upregulation of several target genes, such as genes encoding for inhibitory receptors (*PDCD1* or *LAG3*) or cytokines (*IL32* and *IL21*).

IL12Rβ1G‐β2L, in accordance with STAT4 phosphorylation and IFN‐γ release data, exhibited the strongest upregulation of target genes among all mimetics tested. For some of the target DEGs, upregulation was even more pronounced in comparison to (rh) IL‐12. These targets are related to T‐cell activation (e.g., *IL32*, *IL2RG*, *CGAS*, *ITGAE*, and *CCR1*) or to transcription factors driving effector potential (*TBX21*). Importantly, also inhibitory‐related genes were significantly upregulated by IL12Rβ1G‐β2L treatment in a similar (*SOCS3*, *PDCD1*, and *LAG3*) or even more pronounced fashion (*KLRC1*) when compared with (rh) IL‐12. aDIG_IL12Rβ1G‐β2L, the *C*‐terminal counterpart of IL12Rβ1G‐β2L, was weakened in the elicited transcriptional profile, which is consistent with attenuated maximal STAT4 activation.

While the gene expression profile triggered by IL12Rβ1Y‐β2D (showing highly attenuated potencies in STAT4 phosphorylation) was overall similar (but for most genes not as pronounced) to IL12Rβ1G‐β2L, differences in the transcriptional signature were observed for specific target genes, such as upregulation of *TNFRSF1B*, *IL23R*, and *IL17F*. Essentially, each of the generated IL‐12 mimetics induced an IL‐12‐like but unique transcriptional signature in stimulated T cells.

## 3 DISCUSSION

To preferentially trigger IL‐12R activation on stimulated T cells with limited activation of immune cells in the circulation, we have generated bispecific surrogate agonists by exploiting novel sdAbs which target different epitopes on the IL‐12R subunits. For this, we have isolated camelid‐derived VHH domains using yeast display and phage display after camelid immunization (Valldorf et al. 2021). Single‐domain antibody paratopes, as naturally found in camelids (Könning et al. 2017) and cartilaginous fish (Zielonka et al. 2015), afford the benefit of multiple reformatting options, especially regarding bi‐ and multispecific Ab architectures (Chanier and Chames 2019). In this regard, sdAbs can be readily combined with Fab‐based paratopes in a single molecule (Boje et al. 2024; Lipinski et al. 2023b).

IL‐12 is a pleiotropic molecule primarily acting on T cells and NK cells but also other subsets such as NKT cells or hematopoietic progenitors (Tait Wojno et al. 2019; Trinchieri 2003). Due to the potent proinflammatory nature of this cytokine, IL‐12 emerged as a promising interleukin for therapeutic purposes. However, the clinical value of IL‐12 has been impeded by associated toxicities (Jia et al. 2022; Leonard et al. 1997). In the present study, we were able to engineer sdAb‐based IL‐12 mimetics that displayed distinct biases compared with wild‐type IL‐12 in decoupling the activation of cells isolated from PBMCs from triggering a proinflammatory response in stimulated T cells. In vitro pre‐activated T cells share main characteristics of antigen‐experienced T cells within the tumor microenvironment, such as upregulation of exhaustion markers PD‐1, TIM‐3, LAG‐3, and CTLA‐4 (Andrews et al. 2024; Crawford and Wherry 2009; Lak et al. 2022) as well as enhanced IL‐12Rβ1 expression. Interestingly, the fine epitopes being addressed by the individual sdAbs seemed to be primarily responsible for agonism capacities. This is in line with previous findings of Garcia and co‐workers who were able to highlight the importance of the targeted epitopes on the receptor subunits, ultimately influencing the geometry of the receptor complex (Yen et al. 2022). The group also demonstrated that VHH‐based IL‐2 mimetics can be biased in terms of differentiating naïve T cells into central memory, effector memory, or T cells harboring an exhausted state (Yen et al. 2022).

Besides eliciting divergent activities in terms of STAT4 phosphorylation in NK‐92 cells as well as in a more artificial reporter system using stably transfected IL‐12R overexpressing Ba/F3 cells (Esch et al. 2020) (Figure S10), the four deeply characterized IL‐12 surrogate agonists within our study were also distinct in their ability to preferentially activate stimulated T cells. In fact, two of the generated IL‐12 mimetics, IL12Rβ1G‐β2K and IL12Rβ1U‐β2C were almost inactive on non‐stimulated T cells and NK cells but facilitated moderate STAT4 phosphorylation on pre‐activated T cells. Notwithstanding, both molecules triggered a quite robust release of IFN‐γ on stimulated T cells. Another bispecific surrogate agonist referred to as IL12Rβ1Y‐β2D harbored a unique functionality in a notably different manner. While the tendency for preferential stimulated T‐cell activation was not as pronounced as for IL12Rβ1G‐β2K and IL12Rβ1U‐β2C, this molecule was highly attenuated in terms of potencies, meaning IL12Rβ1Y‐β2D was only active at very high concentrations, rendering this mimetic as a potential candidate for cis‐delivery of a cytokine functionality (Codarri Deak et al. 2022; Zou et al. 2024).

In a previous work by our group, we were able to show that the orientation of individual paratopes as well as valences of individual sdAbs within the overall design architecture have major ramifications regarding signaling strength of engineered IL‐18 mimetics (Lipinski et al. 2023a). This was also demonstrated by Garcia et al. for surrogate agonists mimicking IL‐2 (Yen et al. 2022). Peculiarly, in the present work our findings suggest that the antibody format has also major implications with respect to the cell bias. When IL12Rβ1G‐β2L, which harbored only a minor bias towards stimulated T cells when compared to wild‐type IL‐12, was reformatted from a *N*‐terminal to a *C*‐terminal arrangement (from VHH‐Fc to IgG‐VHH) we observed a considerably augmented predisposition for triggering agonism on pre‐activated T cells. Of note, affinities for binding to the individual receptor subunits were rather similar between both arrangements and also linker lengths of both formats suggested a similar flexibility of engineered designs (Figure 3a). Nevertheless, interference pattern shifts in BLI experiments indicated a slight decrease in binding capacities, likely due to steric hindrance of minor nature for the paratope directed against IL‐12Rβ2, rendering the surrogate agonist in the *C*‐terminal arrangement more susceptible to changes in the expression level of IL‐12Rβ1. When fused to the *C*‐terminus, the CDRs of a given VHH that are primarily responsible for antigen binding, are directed towards the Fc region and receptor binding would require a pronounced conformational bending of the 3xGly_4_Ser linker, which might explain a trend towards slightly reduced binding capacities. This is opposed to the natural *N*‐terminal orientation, where the paratope is facing towards the “outside,” enabling uncompromised epitope binding to constrain IL‐12Rβ1 and IL‐12Rβ2 in a spatial orientation that facilitates robust dimerization and signal transduction. Consequently, it is tempting to speculate that this slightly diminished binding capacity is shifting the agonism bias towards pre‐activated T cells.

Profiling of the transcriptional signatures in stimulated T cells induced by either IL‐12 or the generated surrogate agonists unveiled divergent but IL‐12‐like upregulation of gene expression for the different engineered bsAbs. By specifically taking into consideration DEGs upregulated by IL‐12 itself, we witnessed that IL‐12 mimetics with a stronger induction of STAT4 phosphorylation also triggered the expression of more genes that were identical to IL‐12. A similar observation was made by Garcia and co‐workers for engineered IL‐2 mimetics (Yen et al. 2022). Here, the authors described a linear correlation between pSTAT5 activity and potencies of regulating gene expression. In addition, all selected IL‐12 mimetics generated in this study were capable of inducing transcription of various genes that are known to be upregulated by IL‐12, such as *IFNG*, *IL18R1*, *IL18RAP*. However, differential expression was seen for specific genes, such as *IL32*, *TNFRSF1B*, *TBX21*, *IL23R*, or *IL21*. This was also true for the expression of genes encoding for inhibitory receptors such as *KLRC1*, *PDCD1*, and *LAG3*.

IL‐12 and IL‐23 share the p40 subunit as well as the corresponding common IL‐12Rβ1 chain. Albeit with relatively low affinities, in both cytokines the shared p40 subunit directly interacts with IL‐12Rβ1 (Glassman et al. 2021). Consequently, IL‐12 binding could prevent IL‐23 from IL‐12Rβ1 binding and could attenuate full IL‐23 receptor engagement (and vice versa). To scrutinize whether the herein engineered IL‐12 mimetics share an identical or overlapping epitope with the p40 subunit, we conducted epitope binning experiments with IL‐12 (Figure S2). This revealed non‐competitive binding for all surrogate agonists, indicating different epitopes on IL‐12Rβ1 compared with p40. Thus, in contrast to IL‐12, the engineered surrogate agonists would leave the physiological IL‐23 axis unaffected.

Ultimately, this study is giving evidence that sdAb‐based IL‐12 mimetics are a viable alternative to the engineering of IL‐12 itself (Glassman et al. 2021; Liebl et al. 2023) for the generation of biased agonists with tailor‐made characteristics that differ from wild‐type IL‐12. Besides this particular cytokine, the IL‐12 family consists of several other key members, including IL‐23 or IL‐27, which are crucial in regulating immune responses (Hunter 2005). As outlined above, some of such members share identical receptor subunits with IL‐12. Consequently, sdAbs identified within this study might also function as versatile building blocks for the generation of surrogate agonists mimicking other members of the IL‐12 family.

## 4 METHODS

### 4.1 Camelid immunization

One male llama (*Lama glama*) and one male huarizo (*Lama glama* × *Vicugna pacos*) were immunized with a cocktail (1:1 ratio) of (rh) His‐tagged IL‐12Rβ1 ECD (Acro Biosystems) and Fc‐tagged IL‐12Rβ2 ECD (R&D). The antigen was diluted with sterile deionized water according to manufacturer's recommendation to a final concentration of 200 μg/mL. The animals were immunized subcutaneously with 0.2 mg antigen mixture using 1 mL Gerbu fama adjuvant. The procedure was repeated three times with Incomplete Freund's Adjuvant on Day 14, Day 28, and Day 35. One week after the final administration (Day 43) 100 mL whole blood was collected from each animal for PBMC isolation, RNA isolation, and cDNA synthesis. Animal procedures were conducted at preclinics GmbH, Germany in accordance with local regulations and animal welfare protection laws. Immunized animals remain alive after final blood collection.

### 4.2 Yeast strains and media

For yeast surface display, the *Saccharomyces cerevisiae* strain EBY100 (MATa URA3‐52 trp1 leu2Δ1 his3Δ200 pep4::HIS3 prb1Δ1.6R can1 GAL (pIU211:URA3)) (Thermo Fisher Scientific) was employed. Initially EBY100 were cultivated in yeast extract–peptone–dextrose (YPD) medium composed of 20 g/L peptone, 20 g/L dextrose, and 10 g/L yeast extract supplemented with 0.1 mg/mL penicillin–streptomycin (Gibco). After homologous recombination‐based cloning, cells harboring library plasmids (pDisp) were cultivated in medium using minimal synthetic defined (SD)‐base (Takara Bio) and corresponding dropout mix (Takara Bio) composed of all essential amino acids except for tryptophan (−Trp) for selection, supplemented with 5.4 g/L Na_2_HPO_4_ and 8.6 g/L NaH₂PO₄ × H_2_O. For induction of antibody gene expression, cells were transferred into galactose containing SG dropout medium (−Trp), consisting of SG‐base medium (Takara Bio) supplemented with 10% (w/v) polyethylene glycol 8000 (PEG 8000).

### 4.3 Plasmids for yeast surface display and library generation

Construction of VHH display libraries in yeast was accomplished using homologous recombination‐based gap repair cloning. For this, a detailed protocol described by our group can be found elsewhere (Roth et al. 2020). In brief, amplified VHH library candidates are genetically fused in frame to Aga2p by replacement of a stuffer sequence due to gap repair cloning in the *Bsa*I digested display plasmid pDisp. Full‐length presentation of VHH variants on the yeast cell surface can be detected due to the additional insertion of a HA epitope linked *C*‐terminally to Aga2p on the pDisp backbone.

### 4.4 Library sorting

Initially, EBY100 cells were grown overnight in SD medium with dropout mix lacking tryptophan (−Trp) at 30°C and 120 rpm before inducing VHH surface expression by cell transfer into SG medium with dropout mix (−Trp) at 10^7^ cells/mL for another 48 h at 20°C and 120 rpm. Libraries were sorted individually for each receptor subunit to detect specific antigen binding by incubation with 1 μM either (rh) His‐tagged IL‐12Rβ1 ECD (Acro Biosystems, ILB‐H52H9) or Fc‐tagged IL‐12Rβ2 ECD (R&D, 1959‐B2B), respectively (rh) His‐tagged IL‐12Rβ2 ECD (Acro Biosystems, ILB‐H52H6). Antigen binding was detected in combination with anti‐His mouse monoclonal detection antibody (SureLight® Allophycocyanin, Abcam, ab72579, RRID: AB_1267597, diluted 1:20), Penta His Alexa Fluor 647 Conjugate (QIAgen, 35370, RRID: AB_3083468, diluted 1:20), or rabbit anti‐human IgG Alexa Fluor 647 (Jackson ImmunoResearch, 309‐606‐008, RRID: AB_2339798, diluted 1:100). For library sorting purposes, full‐length surface presentation was simultaneously detected by application of a FITC labeled rabbit polyclonal anti‐HA antibody (Abcam, ab1208, RRID: AB_298835, diluted 1:20) which allowed for a two‐dimensional sorting strategy (Figure S1a,b). The FACS procedure was performed on a BD FACSAria™ Fusion cell sorter (BD Biosciences) device. Untreated cells or cells only incubated with secondary detection reagents or cells incubated with an unrelated antigen served as controls in every experiment allowing for gate adjustment of the desired cell population. After sequencing of enriched populations, after two to three sorting rounds, a clonotyping strategy to cluster the VHHs based on their CDR3 regions was applied as described elsewhere (Lipinski et al. 2023a; Roth et al. 2020).

### 4.5 Phage display

For the construction of phage display libraries, a one‐step Golden Gate Cloning (GGC) approach with type II restriction enzymes was used, to subsequently select for target specific VHHs in three panning rounds. For this, detailed protocols can be found elsewhere described by our group (Bauer et al. 2023; Sellmann et al. 2020). In brief, a destination plasmid carrying an Ampicillin resistance gene for selection and a stuffer region flanked by *Sap*I type II restriction endonuclease recognition sites was digested to incorporate amplified VHH sequences with recognition sites in opposite orientation. After GGC assembly and transformation into electrocompetent *E. coli* cells (Lucigen), the libraries were packaged using M13K07 helper phage (NEB) to produce phage library displaying the VHH diversity. Selection was conducted through three panning rounds, each time beginning with a negative selection against an unrelated protein with the same tag to enrich for target‐specific phages. The target, Fc‐tagged IL‐12Rβ2 ECD (R&D) was immobilized, and non‐bound phages were washed away before elution with trypsin. The output was titrated and used for further rounds, with increasing stringency in the selection process (150, 100, and 50 ng of target antigen). Following the final panning round, individual bacterial clones were screened via ELISA, to detect clones considered hits based on their absorbance readings indicating successful binding to the target antigen (Figure S1c).

### 4.6 Protein expression, purification, and analytics

For the expression of bispecific SEEDbodies (1 + 1, IL‐12Rβ1xIL‐12Rβ2) constructs, selected VHH variants directed against IL‐12Rβ1 ECD were *N*‐terminally fused to the hinge region of Fc immune effector‐silenced (eff‐) SEED AG chains and paired with IL‐12Rβ2‐specific VHHs fused to the N‐terminus of the Fc‐effector silenced GA chain accordingly. For initial binding experiments, respective sdAb SEED chains were paired with a paratope‐less counterpart SEED chain, resulting in monovalent and monospecific SEEDbodies (1 + 0). In addition, VHHs of IL‐12Rβ1G‐β2L were *C*‐terminally fused to an isotype control antibody (aDIG) with a 15 amino acid linker (3xGly_4_Ser). Fc‐effector silencing was introduced by amino acid exchanges L234A and L235A (Schlothauer et al. 2016). Cloning into pTT5 mammalian expression vector (Durocher et al. 2002) enabled protein expression by transient transfection of 5 mL ExpiCHO‐S™ or 25 mL Expi293™ according to the manufacturer's instructions (Thermo Fisher Scientific) using a 2:1 plasmid ratio for SEEDbodies (2xAG:1xGA). Small‐scale production of protein containing supernatants were harvested by centrifugation after 6 days and purified via MabSelect antibody purification chromatography resin (Cytiva). After sterile filtration with Lyzer™‐CL GV 0.22 μm centrifugal devices (Merck Millipore) protein concentrations were measured using Nanodrop ND‐1000 (Peqlab). Protein purities were afterward determined by analytical SEC on a TSKgel UP‐SW3000 column (2 μm, 4.6 × 300 mm, Tosoh Bioscience) using an Agilent HPLC 1260 Infinity system. 7.5 μg protein per sample were injected and run at a flow rate of 0.35 mL/min using 50 mM sodium phosphate, 0.4M NaClO_4_ pH 6.3 as mobile phase.

### 4.7 Biolayer interferometry

For all Biolayer interferometry (BLI) measurements the Octet RED96 system (ForteBio, Pall Life Science) using 25°C and 1000 rpm agitation settings was employed. The data were fitted and analyzed with ForteBio data analysis software 8.0 using a 1:1 binding model after Savitzky–Golay filtering if needed. To evaluate binding to (rh) IL‐12R the respective antigen with His‐tag, either IL‐12Rβ1 ECD (Acro Biosystems) or IL‐12Rβ2 ECD (Acro Biosystems), was loaded on anti‐Penta His (HIS1K) biosensors at 3 μg/mL in PBS for 180 s, followed by 60 s sensor rinsing in kinetics buffer (KB; PBS + 0.1% Tween‐20 and 1% bovine serum albumin, BSA). Association of monospecific IL‐12R SEEDbodies at 100 nM in KB was measured for 180 s. To evaluate binding affinities to each receptor subunit (KD), 3 μg/mL of the constructs were loaded on anti‐hIgG Fc capture (AHC) biosensors 180 s, followed by 60 s sensor rinsing in KB. Association of consecutive dilutions (100 nM, 1:2 dilutions) of IL‐12Rβ1 ECD (Acro Biosystems) or IL‐12Rβ2 ECD (Acro Biosystems) in KB were measured for 300 s followed by dissociation KB for 300 s. Competition of selected agonists in binding to the respective receptor subunit were analyzed in Epitope Binning experiments. HIS1K biosensors were used to load either 5 μg/mL (rh) IL‐12Rβ1 ECD (Acro Biosystems) or IL‐12Rβ2 ECD (Acro Biosystems) in PBS, followed by 60 s sensor rinsing in KB. Association of 200 nM first antibody in 300 s was combined with a second association (300 s) of a second antibody in 200 nM with an included concentration spike of 100 nM of the first used antibody. KB + spike and KB only control values were measured in parallel to ensure visualization of additional association, which was normalized at 300 s. In the same combinatorial manner competition to IL‐12 was analyzed using rh IL‐12 (Acro Biosystems). To compare kinetic constants of agonists IL12Rβ1G‐β2L and aDIG_IL12Rβ1G‐β2L, each construct was covalently immobilized on Amine Reactive Second‐Generation (AR2G) biosensors at 15 μg/mL in PBS for 300 s. To this end, AR2G biosensors were activated by EDC/NHS complex formation and quenched with 1M Ethanolamine after immobilization of antibodies. After antibody immobilization, biosensors were rinsed for 60 s in KB and association of (rh) IL‐12Rβ1 ECD (Acro Biosystems) or IL‐12Rβ2 ECD (Acro Biosystems) with concentrations from 25 to 0.39 nM (in KB) was recorded for 300 s prior dissociation in KB for 300 s. In each experiment, one negative control using an unrelated antibody and a baseline association in KB instead of the respective protein was included.

### 4.8 Cell culture

The parental NK‐92 cell line was cultured in Minimum Essential Medium (MEM) Alpha (Gibco™, 22571), supplemented with 12.5% heat inactivated FBS (Gibco™), 2.5% Horse Serum (Gibco™), and GlutaMAX™ (Gibco™). Cells were passaged twice a week below 0.8 × 10^6^ vc/mL with freshly added 5 ng/mL IL‐2 (Acro Biosystems). Human PBMCs were isolated in‐house from internal healthy blood donors with written informed consent. Whole blood samples were handled according to StemCell Technologies' SepMate PBMC Isolation protocol using SepMate‐50 tubes (StemCell Technologies). PBMCs were stored in DMSO‐containing freezing medium in liquid nitrogen. For experiments, cells were quickly thawed at 37°C and handled in AIM V™ medium (Gibco™). For T‐cell isolation, PBMCs were further processed after isolation using the EasySep™ Human T Cell Isolation Kit (StemCell Technologies). T cells were cultivated using AIM V™ medium (Gibco™) supplemented with 5% AB Serum stimulated depending on experiment conditions with 10 nM IL‐2 and ImmunoCult™ Human CD3/CD28 T Cell Activator (StemCell Technologies). T cells were diluted with fresh medium and respective supplements after 2–3 days. All cells were cultivated at 37°C and 5% CO_2_.

### 4.9 pSTAT4 detection by flow cytometry

For pSTAT4 detection, cells were prepared from culture or frozen stock, washed, and resuspended in respective cultivation medium at 5 × 10^6^ vc/mL. 1 × 10^5^ cells/well were transferred in sterile, round‐bottom, 96‐well plate (Corning). Samples were diluted in the same respective medium and 100 μL of pre‐diluted IL‐12 or IL‐12 variants were added to the appropriate wells. Final concentration of 50 nM IL‐12 was used in control wells to ensure detectable pSTAT4. Plates were then incubated for 45 min at 37°C and 5% CO_2_ in a humidity box. After incubation, cells were centrifuged and washed with pre‐chilled washing buffer (1X PBS, 0.5% BSA) before staining surface marker CD56 labeled with Brilliant Violet 421™ (BioLegend, RRID: AB_2566060). After staining, cells were washed twice with washing buffer and subsequently fixed with addition of 200 μL/well Fixation Buffer (BD Biosciences) by incubation on a plate shaker at 37°C and 350 rpm for 10 min. Then, cells were washed twice again with washing buffer before cell permeabilization by resuspending cells in each well with 150 μL methanol, which was pre‐chilled at −20°C for several hours. Plates were incubated for 30 min on ice. Cells were again washed twice before staining for 45 min with anti‐pSTAT4 (Alexa Fluor® 647, BD Biosciences, RRID: AB_397052) or respective isotype control. After two additional washes, cells were resuspended in 50 μL washing buffer and analyzed on a IQue3 (Sartorius). In experiments involving NK‐92 cells, cells were directly fixated after sample incubation.

### 4.10 IFN‐γ release assay

For investigation of secretion levels of IFN‐γ the human IFNγ (Revvity) HTRF kit was used. The assays were carried out following the manufacturer's protocol. After 24 h of sample stimulation and plate centrifugation, 8 μL of supernatant was directly transferred to 2 μL antibody mixture in 96‐well white low volume HTRF plates (Revvity). The standard was prepared with the respective medium and plates were sealed and incubated overnight at RT. The signal was measured using PHERAstar FSX (BMG LABTECH) and data were analyzed by MARS software (v.3.32; BMG Labtech), enabling a four‐parameter logistic (4PL 1/y2) model fitting of the standard curve. IFN‐γ levels were displayed after deduction of cells incubated without stimulation.

### 4.11 Surface staining by flow cytometry

All washes and dilutions of cells and samples were performed using flow buffer (1X PBS, 1% BSA). Cells were seeded at 1 × 10^5^ cells/well in round‐bottom 96‐well plate (Corning) and handled at 4°C or on ice for entire experiment duration. Cells were washed and incubated for 30 min separately in following detection antibodies: anti‐PD‐1 (PE, BD, RRID: AB_2916671, dilution 1:20), anti‐LAG‐3 (PE, BD, RRID: AB_2571727, dilution 1:20), anti‐TIM‐3 (Alexa Fluor™ 488, BD, RRID: AB_2916395, dilution 1:20), anti‐CTLA‐4 (E‐Cy7, BioLegend, RRID: AB_2563098, dilution 1:20) as well as anti‐IL‐12Rβ1 (PE, Miltenyi Biotech, RRID: AB_2656318, dilution 1:4) and IL‐12Rβ2 (PE, Miltenyi Biotech, RRID: AB_2751988, dilution 1:4) and all respective isotype controls. Following two more washing steps, cells were resuspended in a final volume of 50 μL/well flow buffer with SytoxRed (Invitrogen) for dead cell staining. Cells were analyzed using the IQue3 system (Sartorius). Geometric mean fluorescence intensity was normalized and plotted as a fold over the staining isotype control.

### 4.12 Molecular modeling, structural visualization, and engineering

Structural models of the VHH domains and constant regions of the different bispecific formats were generated using the antibody modeler tool in the molecular modeling software package moe (Molecular Operating Environment 2020.09: Chemical Computing Group Inc.; 2020). VHHs domains were either directly fused to the constant regions or added via a Gly_4_Ser‐linker using moe's protein builder, followed by a conformational search of the linker and an energy minimization of the full constructs. Visualization of 3D structures and properties was done with PyMOL (The PyMOL Molecular Graphics System, Version 2.3.0 Schrödinger, LLC.).

### 4.13 RNA sequencing

Isolated T cells were either rested 1 day after isolation or pre‐activated for 7 days by CD3/CD28 and IL‐2 as described. Cells were washed with respective medium and seeded in 96‐well plates at 1 × 10^6^ cells/well, then stimulated with 50 nM IL‐12 or surrogate agonists for 24 h at 37°C. Total RNA was extracted of each condition from three independent donors using a RNeasy Plus Mini kit (Qiagen). cDNA library preparation and RNA sequencing were performed by Novogene using an Illumina NovaSeq 6000 sequencer, PE150 platform. Analysis from Novogene with reference genome (*Homo Sapiens* [GRCh38/hg38]) and differential expression analysis was further evaluated using Novomagic (after‐sales analysis cloud platform independently developed by Novogene).

### 4.14 Generation of Ba/F3 cells with human IL‐12 receptors

Ba/F3 (ACC 300) cells were procured from the Leibnitz Institute DSMZ‐German Collection of Microorganisms and Cell Culture (Braunschweig, Germany) and retrovirally transduced with pMOWS expression plasmid coding for hIL‐12Rβ1‐F2A‐hIL‐12Rβ2 as previously described in Esch 2020 (Esch et al. 2020). The transduced cells were cultivated in Dulbecco's modified Eagle medium (DMEM) with a high glucose concentration (Gibco™) and 10% fetal bovine serum (Gibco™). The medium was further enriched with 60 mg/L penicillin and 100 mg/L streptomycin (Genaxxon bioscience GmbH) and 0.2% conditioned cell culture supernatant of IL‐3 secreting WEHI‐3B cells. Transduced Ba/F3 cells were then selected with puromycin (1.5 mg/mL) (Carl Roth GmbH, Karlsruhe, Germany) for a period of at least 2 weeks.

For detection of cell surface expression of the cytokine receptors, stably transduced Ba/F3 cells were washed with FACS buffer (PBS containing 1% BSA) and incubated at 5 × 10^5^ cells/50 μL FACS buffer supplemented with antibodies against human IL‐12Rβ1 (anti‐hIL‐12Rβ1 PE conjugated, R&D Systems, FAB839P, RRID: AB_2124034), or rat anti‐hIL‐12Rβ2 (BD Pharming, 550722, RRID: AB_393850) for 1 h. Subsequent to a single wash with FACS buffer, cells were incubated in 50 μL of FACS buffer containing Alexa Fluor 647–conjugated Fab goat anti‐rat IgG (Jackson ImmunoResearch Labs, 112‐607‐003, RRID: AB_2338413) for 1 h. Finally, cells were washed with FACS buffer and analyzed by flow cytometry (BD FACSCanto II flow cytometer using the FACSDiva software, BD Biosciences). Data were analyzed using the FCS Express software (De Novo Software, Los Angeles, CA).

### 4.15 Analysis of cellular proliferation

Ba/F3 cells were washed three times in sterile phosphate‐buffered saline (PBS) and adjusted to 5 × 10^3^ cells in 100 μL DMEM that was supplemented with 10% FCS, 60 mg/L penicillin, and 100 mg/L streptomycin. Ligands were added in increasing concentrations ranging from 0.00005 to 100 nM, and the cells were incubated for 3 days at 37°C. The CellTiter‐Blue Cell Viability Assay (Promega) was utilized to estimate viable cells by recording the fluorescence (excitation 560 nm, emission 590 nm) using the Infinite M200 PRO plate reader (Tecan) immediately after adding 20 μL of reagent per well (time point 0) and up to 2 h after incubation under standard cell culture conditions. The fluorescent signal from the CellTiter‐Blue Reagent is directly proportional to the number of viable cells. All values were measured in quadruplicates per experiment, and the fluorescence values were normalized by subtraction of the time point 0 values. All experiments were performed at least four times, and one representative experiment was selected for presentation. Determination of the median effective concentration (EC_50_) was conducted through the implementation of a nonlinear regression analysis, incorporating a variable slope calculation within the GraphPad Prism 8.0 (version 8.0.2 for Windows, GraphPad Software, www.graphpad.com) software. Obtained data are presented as the mean ± standard deviation (SD).

### 4.16 Analysis of intracellular signal transduction

Ba/F3‐IL‐12Rβ1‐IL‐12Rβ2 cells were washed three times with PBS and subsequently starved in serum‐free medium for a period of 4 h. Thereafter, cells were stimulated with the indicated ligands for 30 min, harvested by centrifugation at 4°C for 5 min at 1500 rpm and frozen. Cells were lysed for a period of 2 h using a buffer composed of 10 mM Tris–HCl, pH 7.8, 150 mM NaCl, 0.5 mM EDTA, 0.5% Nonidet P‐40, 1 mM sodium vanadate, 10 mM MgCl2, and one complete EDTA‐free protease inhibitor mixture tablet (Roche Diagnostics). Protein concentration of the cell lysates was determined by the bicinchoninic acid Protein Assay (Pierce, Thermo Fisher Scientific). Lysed proteins were then mixed with (5x) SDS loading buffer (125 mM Tris–HCl pH 6.8, 50% glycerol, 10% SDS, 5% β‐mercaptoethanol, bromophenol blue) and incubated at 95°C for 10 min. Separation of proteins was conducted via SDS‐PAGE, followed by their transfer to polyvinylidene difluoride (PVDF) membranes for 60 min (20 V, 1 A). Membranes were then blocked in 5% fat‐free dried skimmed milk in TBS‐T (10 mM Tris–HCl pH 7.6, 150 mM NaCl, 1% Tween 20) and probed overnight with the indicated primary antibodies (1:1000). Following antibodies were obtained from Cell Signaling Technology: phospho‐STAT3 ((Tyr705) (D3A7), #9145, RRID: AB_2491009), STAT3 ((79D7), #4904, RRID: AB_331269), phospho‐p44/42 MAPK (Erk 1/2) ((Thr202/Tyr204), #4370, RRID: AB_2315112), p44/42 MAPK (Erk 1/2) antibody (#9102, RRID: AB_330744). The anti‐γ‐tubulin antibody (#T5326, RRID: AB_532292) was procured from Sigma Aldrich. After washing, membranes were incubated with secondary peroxidase–conjugated antibodies (1:2000, goat anti‐rabbit IgG (H + L) cross‐adsorbed secondary antibody (#31462, RRID: AB_228338) or rabbit anti‐mouse IgG F(ab′)2 secondary antibody (#31451, RRID: AB_228433)) from Thermo Fisher Scientific, Waltham, MA. Immobilon Western Reagents (Millipore Corporation) and the ChemoCam Imager (INTAS Science Imaging Instruments GmbH) were used for signal detection.

### 4.17 Data processing and statistical analysis

Graphical and statistical analyses were conducted with GraphPad Prism version 10.2.1 for Windows, GraphPad Software, Boston, MA (www.graphpad.com). Dose‐dependent curves were fitted using a nonlinear regression curve with three or four parameters, depending on goodness of fit, to calculate *E*_max_, EC_50_ values and 95% confidence intervals. *p*‐values were calculated utilizing appropriate ANOVA analyses followed by Bonferroni test as recommended. *p* ≤ 0.05 was regarded as statistically significant.

## AUTHOR CONTRIBUTIONS

**Britta Lipinski:** Investigation; methodology; data curation; writing – review and editing; writing – original draft; formal analysis. **Laura Unmuth:** Investigation; writing – review and editing; validation; methodology; formal analysis; data curation. **Paul Arras:** Investigation; methodology. **Ron Endruszeit:** Investigation; methodology. **Stefan Becker:** Supervision; validation; writing – review and editing. **Jonathan Mathias Rödel:** Investigation. **Jürgen Scheller:** Methodology; validation; writing – review and editing; formal analysis; data curation. **Silke Pudewell:** Investigation; validation; visualization; methodology; data curation. **Doreen M. Floss:** Methodology; validation; visualization; investigation; writing – review and editing. **Simon Krah:** Writing – review and editing; software; methodology. **Julia Harwardt:** Methodology; validation; visualization; writing – review and editing; formal analysis; data curation. **Achim Doerner:** Writing – review and editing; validation. **Laura Helming:** Writing – review and editing; validation; methodology. **Chunxiao Xu:** Methodology; validation; writing – review and editing. **Andreas Menrad:** Writing – review and editing; validation; methodology; supervision; resources. **Andreas Evers:** Visualization; validation; methodology; software; formal analysis; data curation. **Harald Kolmar:** Supervision; data curation; formal analysis; writing – review and editing. **Desislava Elter:** Investigation; validation; visualization; formal analysis; writing – review and editing. **Lukas Pekar:** Conceptualization; investigation; methodology; validation; visualization; writing – review and editing; formal analysis. **Stefan Zielonka:** Conceptualization; investigation; writing – original draft; project administration; supervision; resources; software; formal analysis; data curation; methodology; validation; visualization.

## CONFLICT OF INTEREST STATEMENT

BL, LU, PA, SB, SK, JH, AD, LH, CX, AM, AE, DE, LP, and SZ are employees at either Merck Healthcare KGaA or EMD Serono. Besides, this work was conducted in the absence of any further commercial interest.

## Supporting information


**Data S1.** Supporting Information.

## Data Availability

The data that supports the findings of this study are available in the supplementary material of this article.
