# Supplementary material for: Taming interleukin‐12: Engineering of bispecific antibody‐based IL‐12 mimetics with biased agonism capacities
Source: Protein Sci. 2025 Feb 21;34(3):e70072. doi: 10.1002/pro.70072 (PMC11843475; doi:10.1002/pro.70072)
Supplement: Supplementary file 1 — Data S1. Supporting Information. [file PRO-34-e70072-s001.docx]

**Supplementary Information: Taming Interleukin-12: Engineering of bispecific antibody-based IL-12 mimetics with biased agonism capacities**

Britta Lipinski^1,2+^, Laura Unmuth^1,2+^, Paul Arras^1^, Ron Endruszeit^1^, Stefan Becker^2^, Jonathan Mathias Rödel^3^, Jürgen Scheller^3^, Silke Pudewell^3^, Doreen M. Floss^3^, Simon Krah^2^, Julia Harwardt^2^, Achim Doerner^2^, Laura Helming^4^, Chunxiao Xu^4^, Andreas Menrad^2^, Andreas Evers^2^, Harald Kolmar^5^, Desislava Elter^2^, Lukas Pekar^2^, and Stefan Zielonka^1,2*^

^1^Biomolecular Immunotherapy, Institute for Organic Chemistry and Biochemistry, Technical University of Darmstadt, D-64287 Darmstadt

^2^Antibody Discovery and Protein Engineering, Merck Healthcare KGaA, D-64293 Darmstadt, Germany

^3^Institute of Biochemistry and Molecular Biology II, Medical Faculty and University Hospital Düsseldorf, Heinrich-Heine-University Düsseldorf, D-40225 Düsseldorf, Germany

^4^Research Unit Oncology, EMD Serono Research Center, Billerica, MA 01821, USA

^5^Applied Biochemistry, Institute for Organic Chemistry and Biochemistry, Technical University of Darmstadt, D-64287 Darmstadt

^+^These authors contributed equally to this work.

^*^To whom correspondence should be addressed:

Stefan Zielonka, Biomolecular Immunotherapy, Institute for Organic Chemistry and Biochemistry, Technical University of Darmstadt, Peter-Grünberg-Straße 4, D-64287 Darmstadt, Germany, E-mail: [stefan.zielonka@tu-darmstadt.de](mailto:stefan.zielonka@tu-darmstadt.de)

**Running title: Biased bispecific IL-12 surrogate agonists**

**
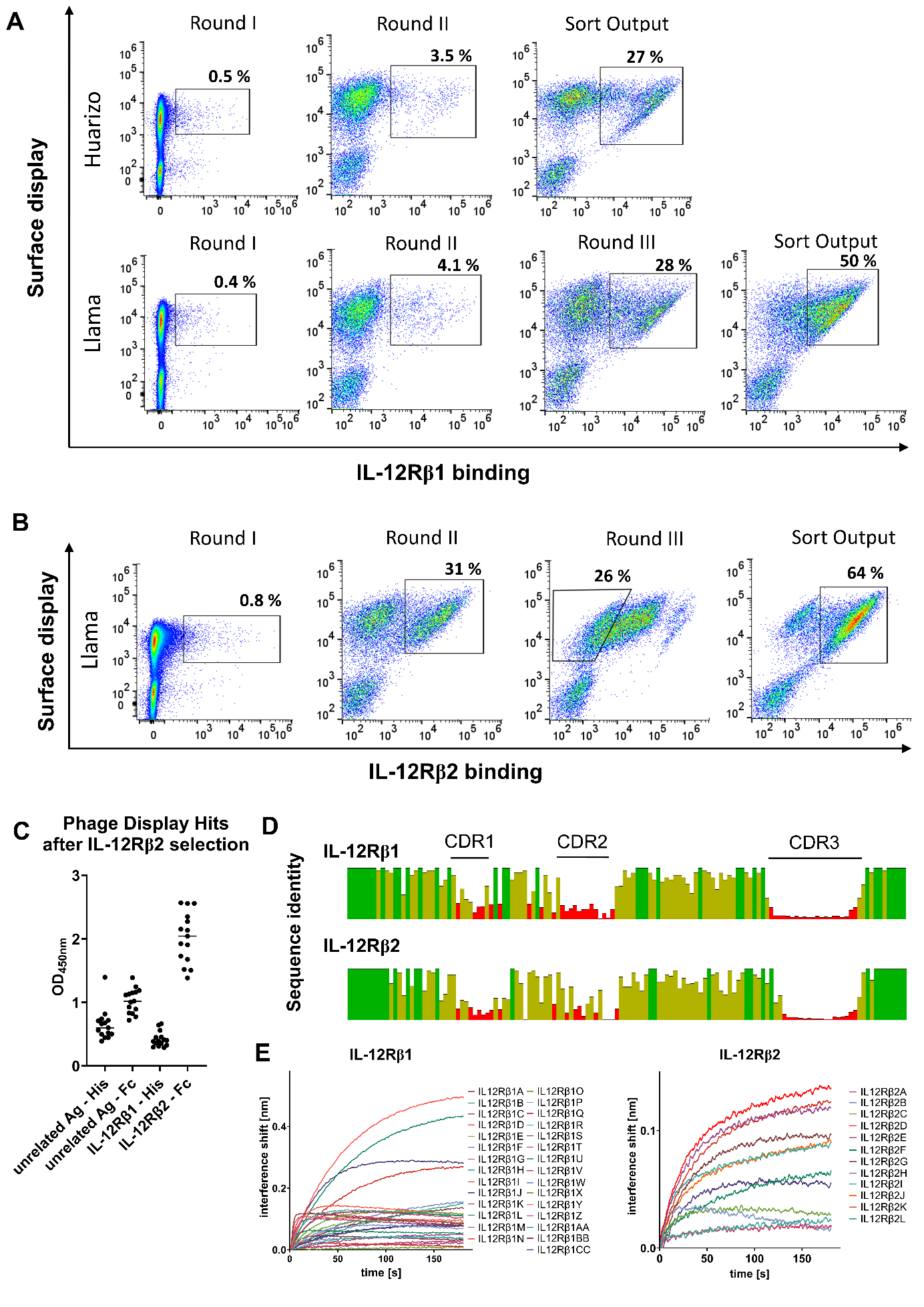
**

**Figure S1: Generation of camelid-derived and IL12R-specific sdAbs based on yeast surface display and phage display selection.** (A) Enrichment of IL-12Rβ1 binding VHHs displayed on yeast libraries, that were generated based on two camelids after immunization. Each antigen was exploited at a concentration of 1 µM throughout all rounds of sorting. A two-dimensional sorting strategy was applied to select for full-length VHH display in addition to binding functionality. Percentage of cells in sorting gates are shown. (B) Library sorting of one specimen (llama) was conducted to enrich for IL-12Rβ2-specific sdAbs which involved the utilization of the (rh) Fc-tagged ECD of IL-12Rβ2 in Round I and the His-tagged counterpart in Round II. In Round III of sorting, the incubation was conducted with an unrelated Fc-tagged antigen. To deplete for VHH domains targeting the human IgG1 Fc part, the sorting gate was adjusted to select for clones displaying the VHH entity but not showing binding against the unrelated Fc fusion protein, resulting in the enrichment of clones specific to the his-tagged ECD of (rh) IL-12Rβ2. (C) Antigen ELISA of 15 single clones after phage display selection for (rh) IL-12Rβ2 binding VHHs. (D) Graphical alignment of 39 unique clones addressing (rh) IL-12Rβ1 and 50 unique clones targeting (rh) IL-12Rβ2 that were selected after sequencing of hits generated *via* selections methods described above. Complementarity-determining regions (CDRs) are highlighted. Red bars indicate high sequence diversity at the amino acid level and green bars represent high sequence conservation at a given position. Alignment conducted with Clustal Omega alignment tool using Geneious version 2023.1. (E) Monospecific (1+0) constructs, which were produced by exploiting strand-exchange engineered domain (SEED) technology, were analyzed by BLI for binding to either IL-12Rβ1 or IL-12Rβ2. For this, the his-tagged ECD of the receptor subunit was loaded to HIS1K biosensors at 3 µg/mL followed by association of sdAb constructs at 100 nM for 180 s.


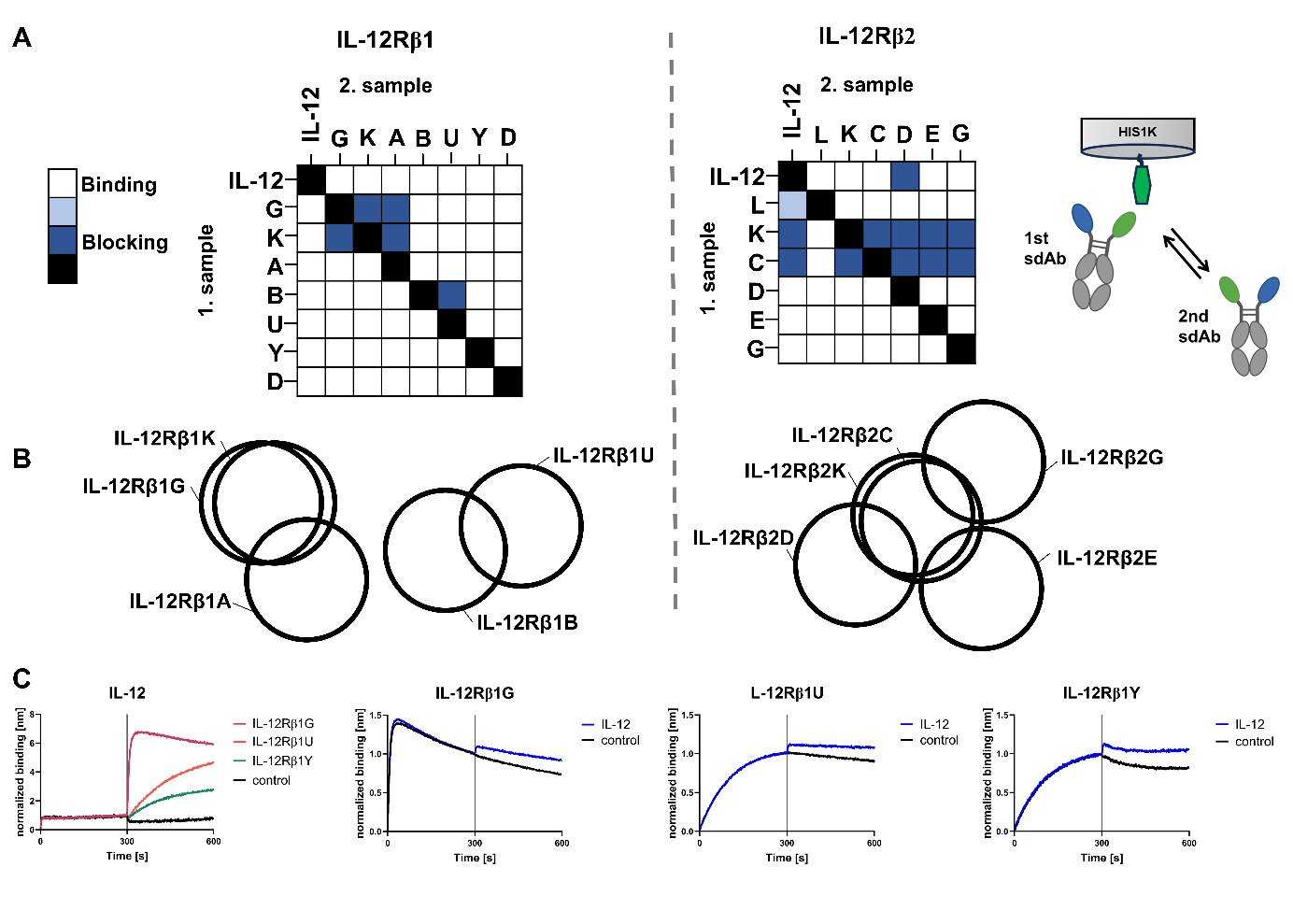


**Figure S2: Epitope binning experiments for binding to IL-12R subunits of TOP10 related paratopes.** (A) Heatmaps display results of BLI-based competition assays of pairwise binding, starting with first association of samples listed in rows with subsequent second association of samples listed in columns. Self-blocking is visualized in black, while blocking of the second associated sample is colored in dark blue. Binding of the second paired sample is highlighted in white. (B) Blocked binding to either (rh) IL-12Rβ1 or (rh) IL-12Rβ2 in both pairwise combinations is visualized by strongly overlapping circles, indicating one presumed epitope bin. Blocking in one direction with an additional association in the other way is depicted by circles with only a small overlap to suggest steric hindrance or an epitope in close proximity impairing second association. (C) Left: Example of non-overlapping epitopes after IL-12Rβ1 loading as demonstrated by two consecutive association steps for IL-12 followed by second association of IL-12Rβ1G, IL-12Rβ1U or IL-12Rβ1Y. Second from left to right: Additional association of IL-12 after binding of IL-12Rβ1G, IL-12Rβ1U or IL-12Rβ1Y to IL-12Rβ1 indicates non-competitive VHHs to the wild-type cytokine in both settings.


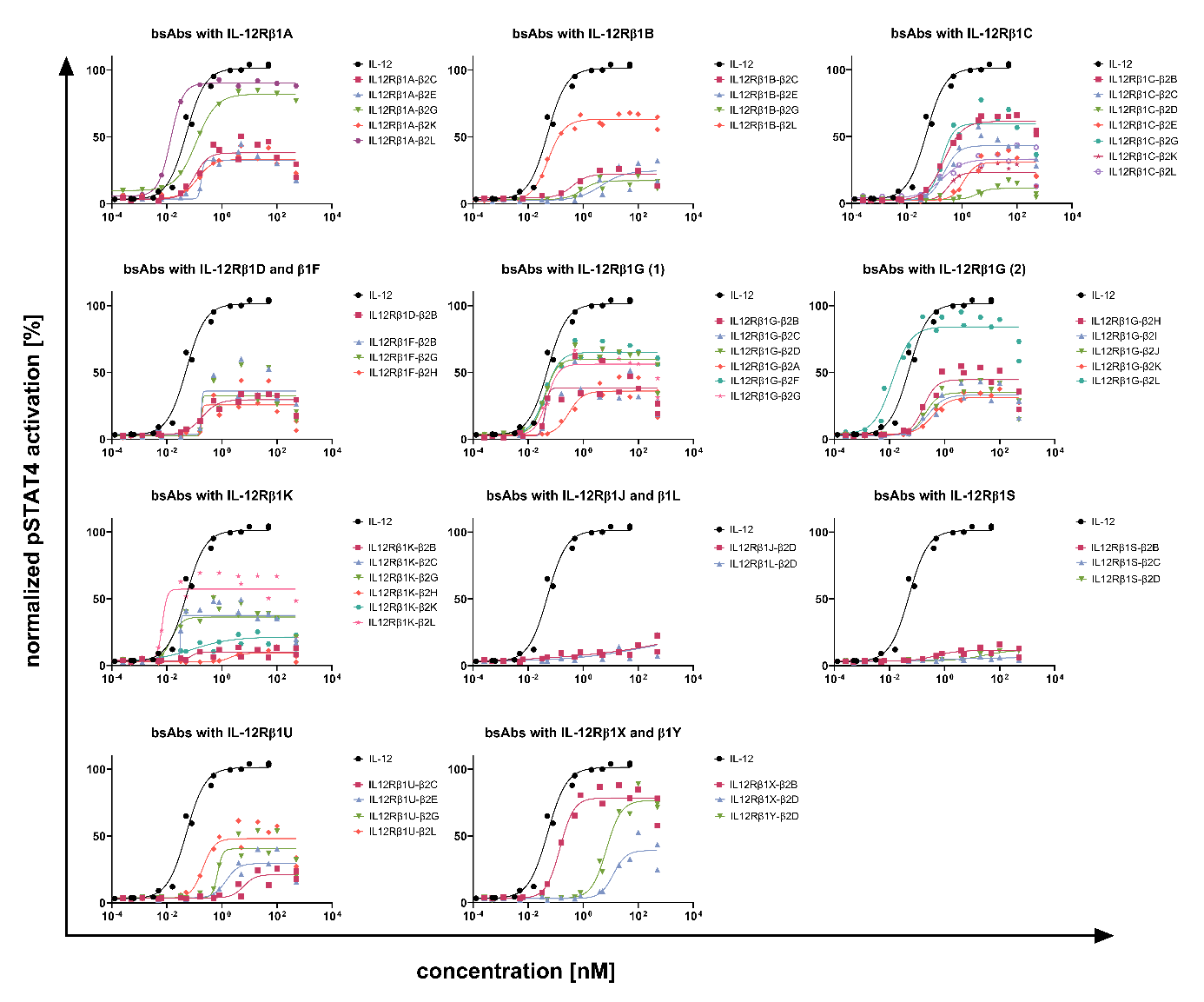


**Figure S3: Combinatorial reformatting to monovalent (1+1) bsAbs enables the identification of IL-12 surrogate agonists activating phosphorylation of STAT4 in a dose-dependent manner.** NK-92 cells were incubated with titrated samples, starting at 500 nM (1:10 and 1:5 dilution steps, n=1-2) and IL-12 as positive control with 50 nM. Values of pSTAT4^+^ cells were normalized to 50 nM IL-12 of the corresponding plate.


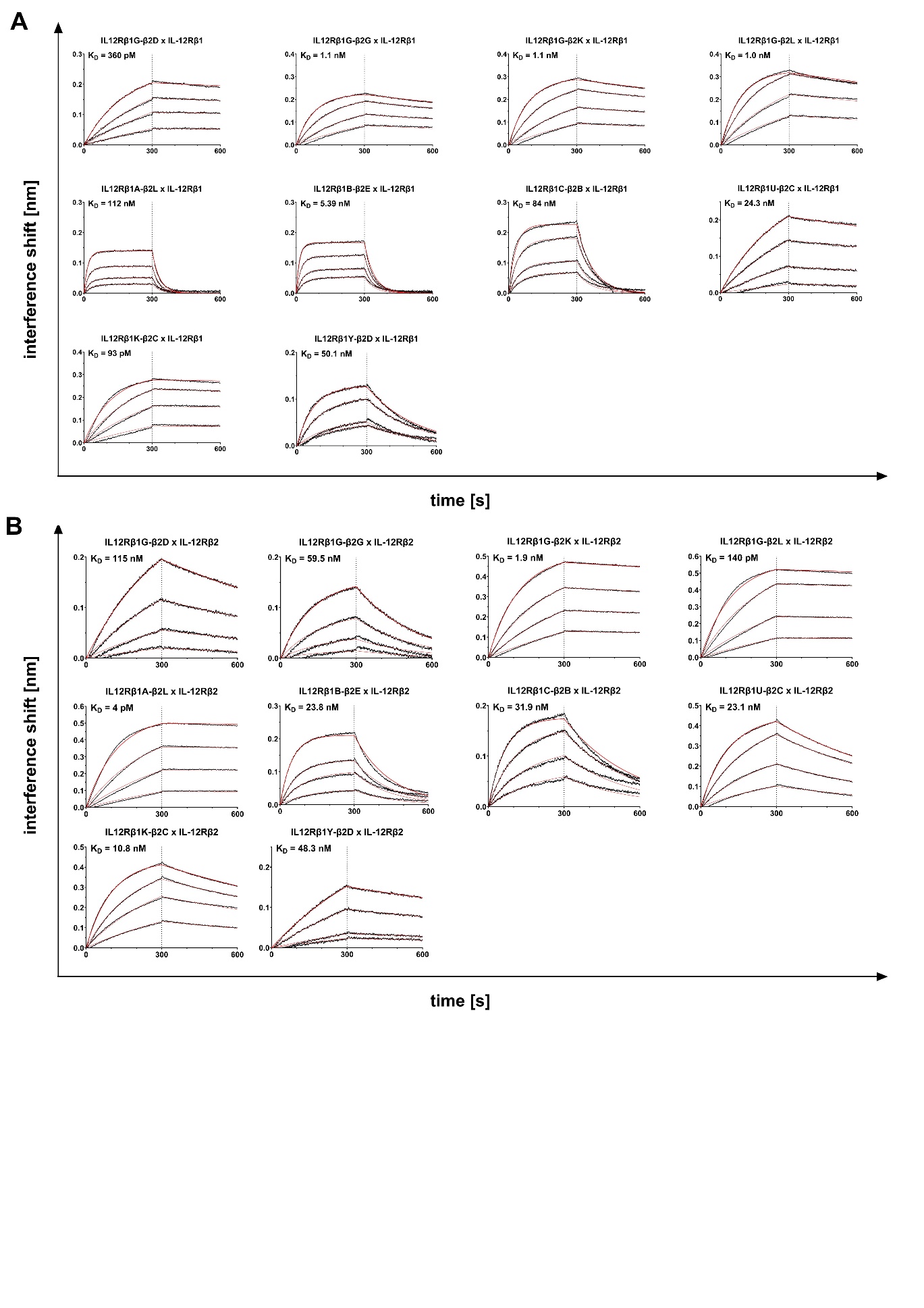


**Figure S4: Binding kinetics of bispecific (1+1) TOP10 IL-12 mimetics against receptor subunits (rh) IL-12Rβ1 and (rh) IL12Rβ2.** IL-12 bsAbs were loaded on AHC biosensors at a concentration of 5 µg/mL and consecutive association with increasing concentrations ranging from 6.25 nM to 200 nM of either IL-12Rβ1 or IL-12Rβ2 for 300 s, followed by dissociation in kinetics buffer for 300 s.

**
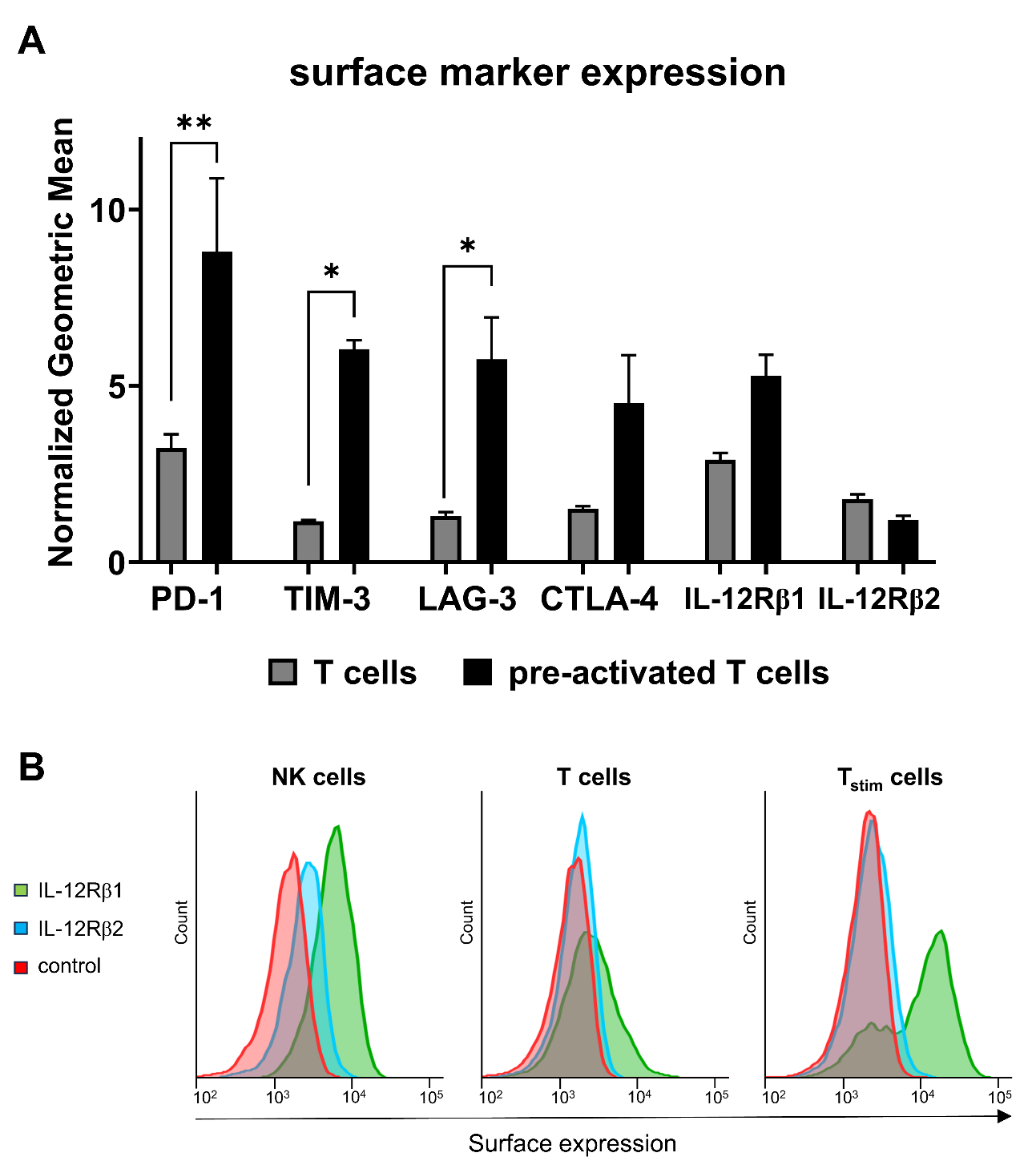
**

**Figure S5: Surface expression profile of exhaustion markers and IL-12Rβ1 as well as IL-12Rβ2 upon T cell stimulation. (**A) Expression levels of T cell exhaustion markers and IL-12R subunits were determined by staining of PD-1, LAG-3, TIM-3 and CTLA-4, as well as IL-12Rβ1 and IL-12Rβ2 for 45 minutes. Isolated T cells with or without 7-day-stimulation of four different donors were stained with detection antibody as well as the respective isotype control to which the geometric mean was normalized. Mean values ± SEM of four independent experiments and healthy donors are displayed. (B) Flow cytometry analysis of IL-12Rβ1 and IL-12Rβ2 on the surface of NK, T and stimulated T cells. Red areas indicate isotype control on respective cells with blue indicating IL-12Rβ2 and green IL12Rβ1 expression. One representative experiment out of three (NK cells) to four biological replicates is shown.


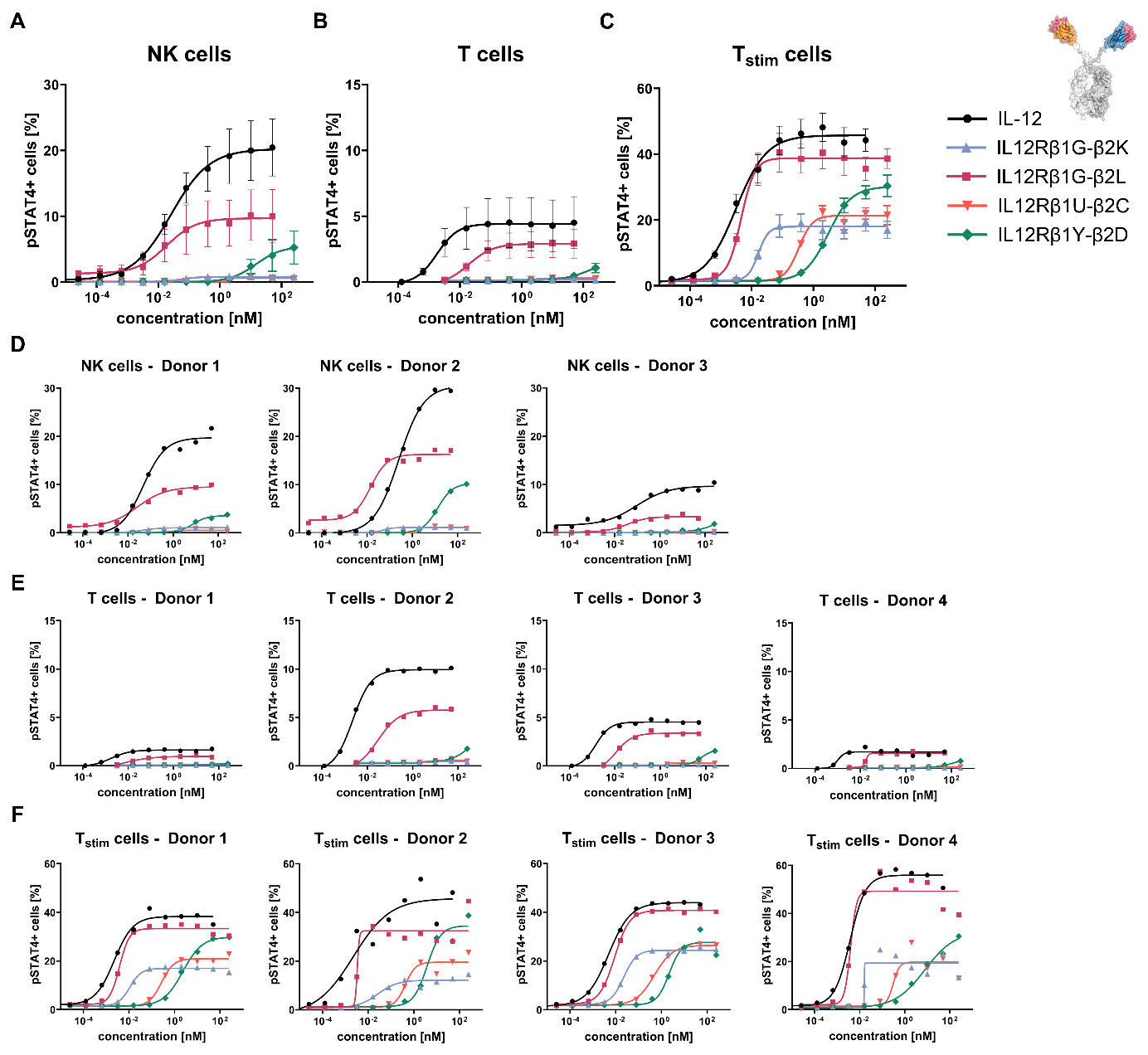


**Figure S6: IL-12 mimicking sdAb-based surrogate agonists preferentially activate stimulated T cells with reduced activity on NK and T cells.** (A, B and C) Zoomed-in view of intracellular staining of pSTAT4-positive cells after 45 min incubation with IL-12 or IL-12 surrogate agonists (IL12Rβ1G-β2K, IL12Rβ1G-β2L, IL12Rβ1U-β2C or IL12Rβ1Y-β2D). Graphs display mean values ± SEM of three (NK cells) to four independent experiments. (D, E and F) Individual graphs of three donors tested for pSTAT4^+^ NK cells and four donors of isolated T cells for pSTAT4-positive cells after 45 min incubation with IL-12 or IL-12 surrogate agonists.


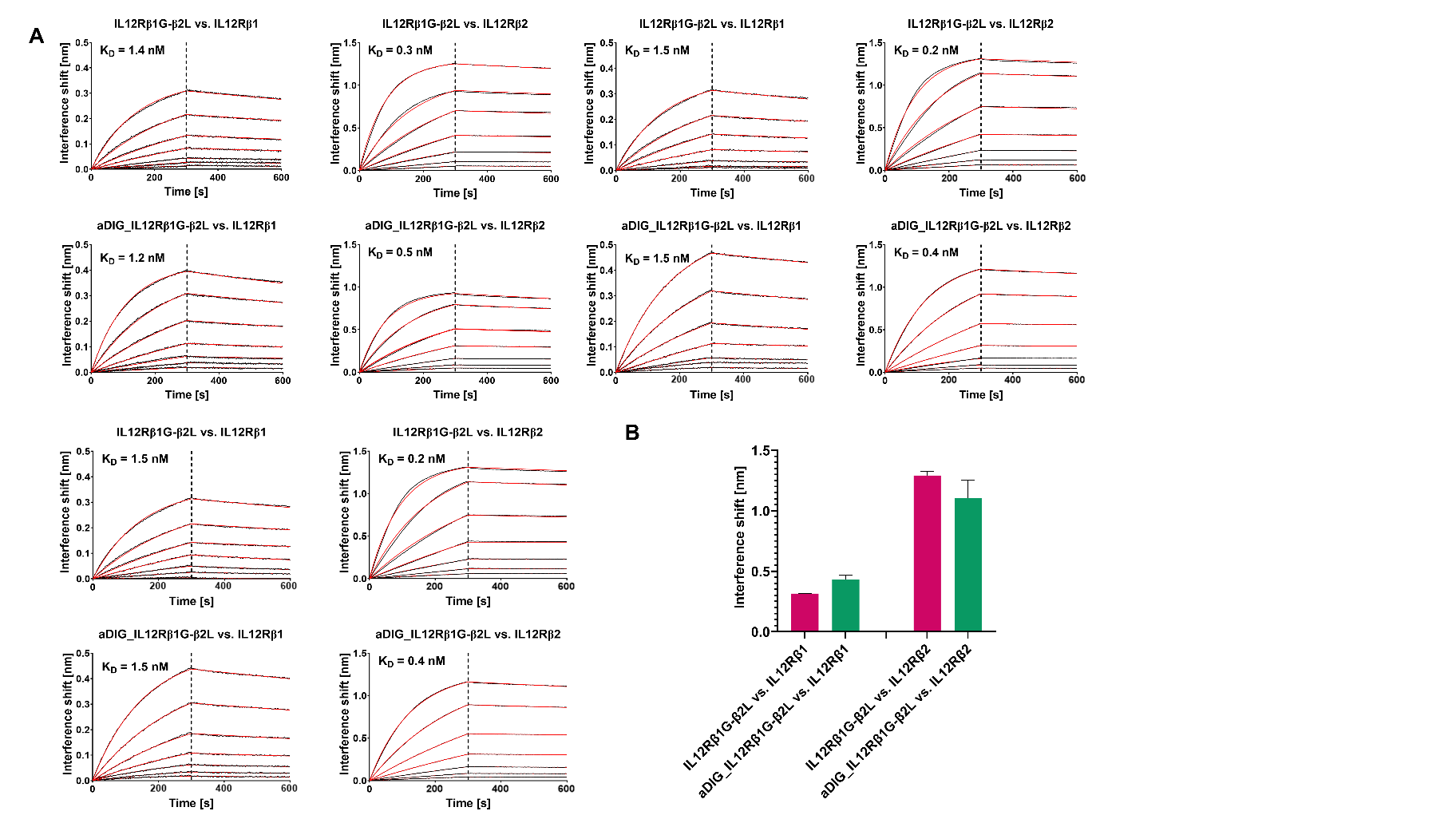


**Figure S7: Binding kinetics of *N*-terminal *vs* *C*-terminal fused VHHs against receptor subunits IL-12Rβ1 and IL12Rβ2 via AR2G biosensors. (A)** Activated AR2G biosensors by EDC/NHS complex formation were immobilized with 15 µg/mL respective antibodies IL12Rβ1G-β2L and aDIG_IL12Rβ1G-β2L and quenched with 1 M Ethanolamine. Association for 300 s of increasing concentrations starting at 0.39 nM to 25 nM of either IL-12Rβ1 or IL-12Rβ2 followed by dissociation in kinetics buffer for 300 s is shown for three individual experiments. (B) Maximum interference shift in nm are depicted for N-terminal and C-terminal fused VHHs against both IL-12 receptor subunits of three independent experiments.


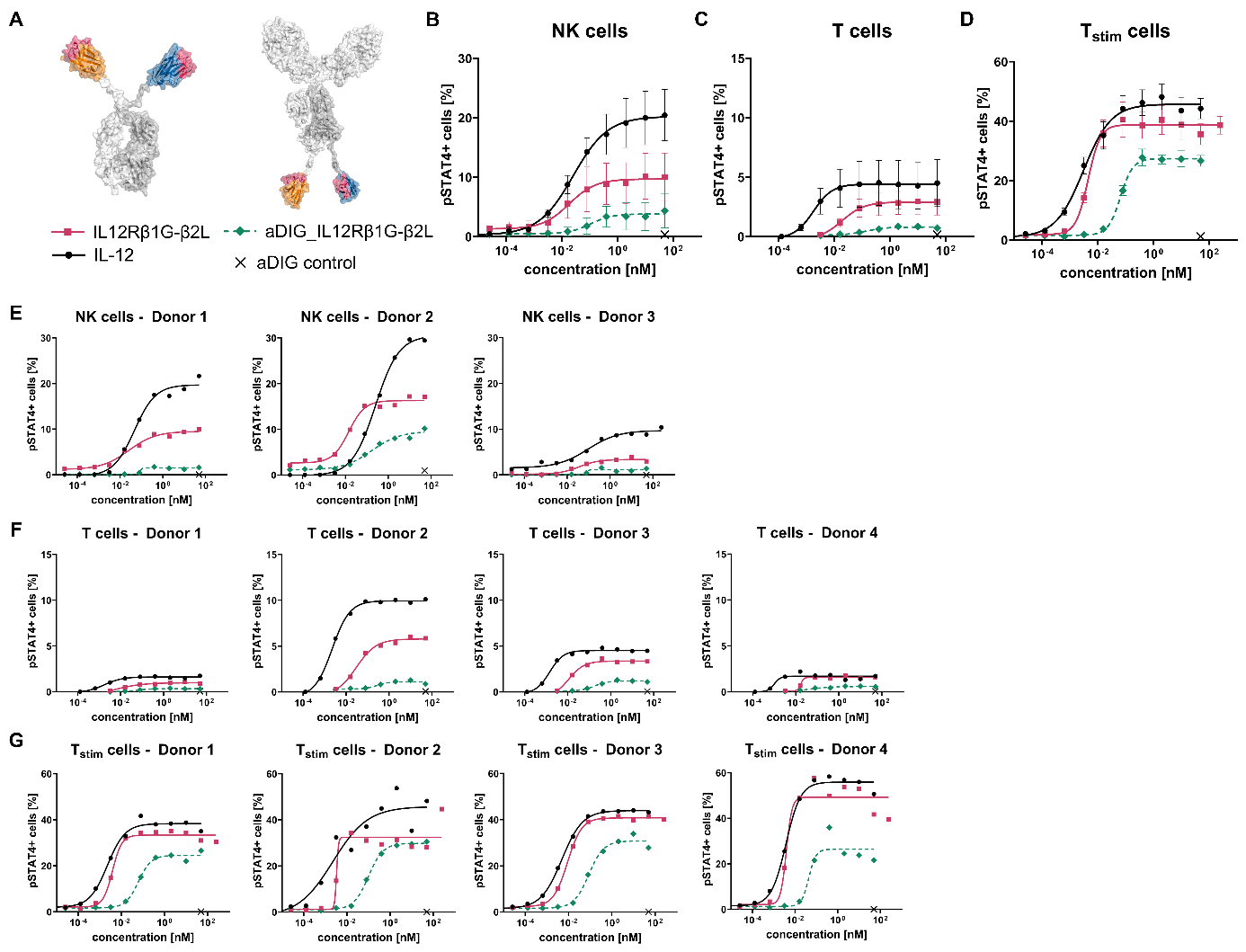


**Figure S8: Antibody engineering enables generation of IL-12 mimetic with pronounced bias for activated T cells.** (A) Scheme of two antibody architectures exploited in this study. (B, C and D) Zoomed-in view of intracellular staining of pSTAT4^+^ cells after 45 min incubation with IL-12 or IL-12 surrogate agonists at different concentrations (IL12Rβ1G-β2L and aDIG_ IL12Rβ1G-β2L). Graphs display mean values ± SEM of three (NK cells) to four independent experiments. (E, F and G) Individual graphs of three donors tested for pSTAT4^+^ NK cells and four donors of isolated T cells for pSTAT4-positive cells after 45 min incubation with IL-12 or IL-12 surrogate agonists.

**
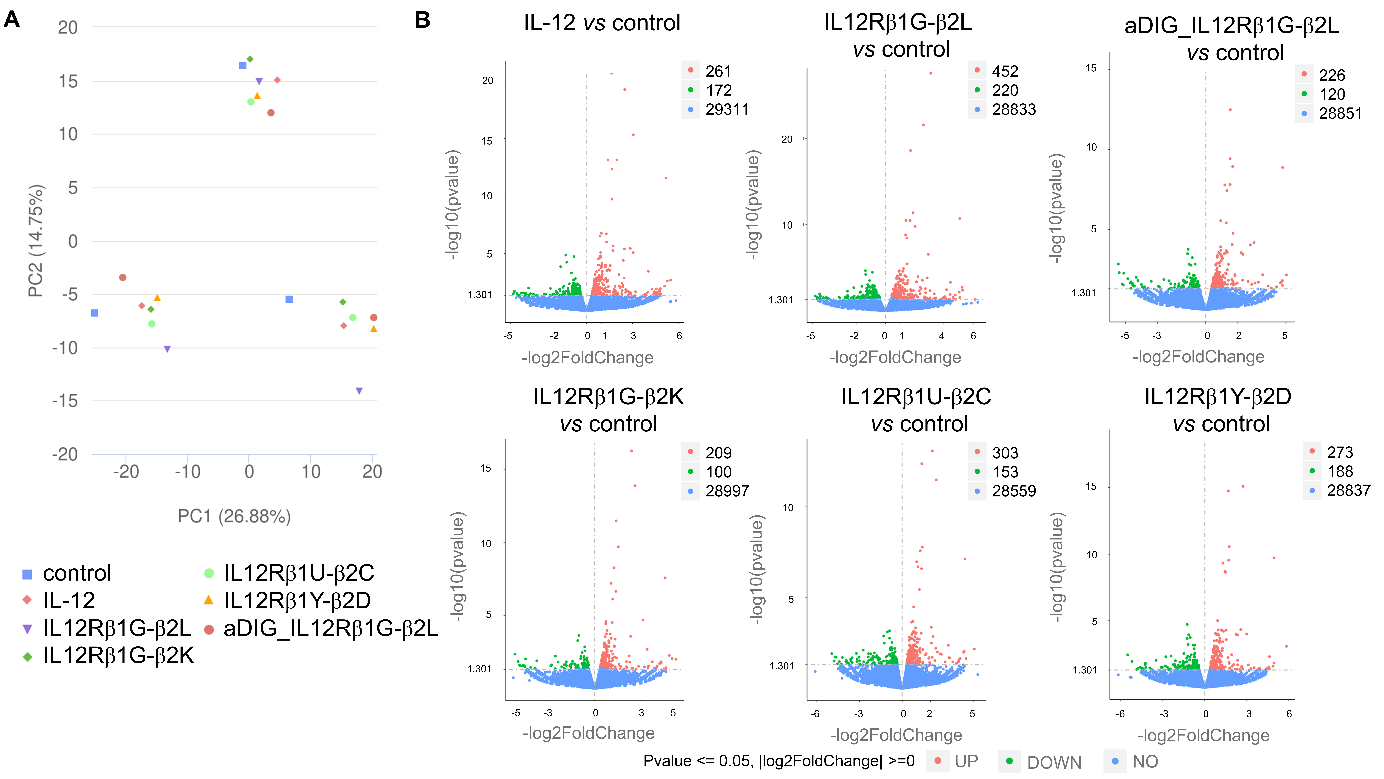
**

**Figure S9: Similarity in gene expression between surrogate agonists and IL-12.** (A) Principal component analysis (PCA) of gene expression in isolated T cells from 3 donors stimulated with IL-12 or surrogate agonists for 24 h. Percentage of variance is described by principal component axes PC1 and PC2. All stimulations of each donor are clustered together due to individual resemblance. Effect of IL-12-like stimulation can be determined by the distance between agonists to the respective control (blue square). (B) Log2-Fold change of genes (padj < 0.05) of every stimulation condition is displayed in the respective volcano plot. Upregulated differentially expressed genes (DEGs) are depicted in red, downregulated DEGs are depicted in green, while not significantly altered genes are visualized in blue below the significance threshold.


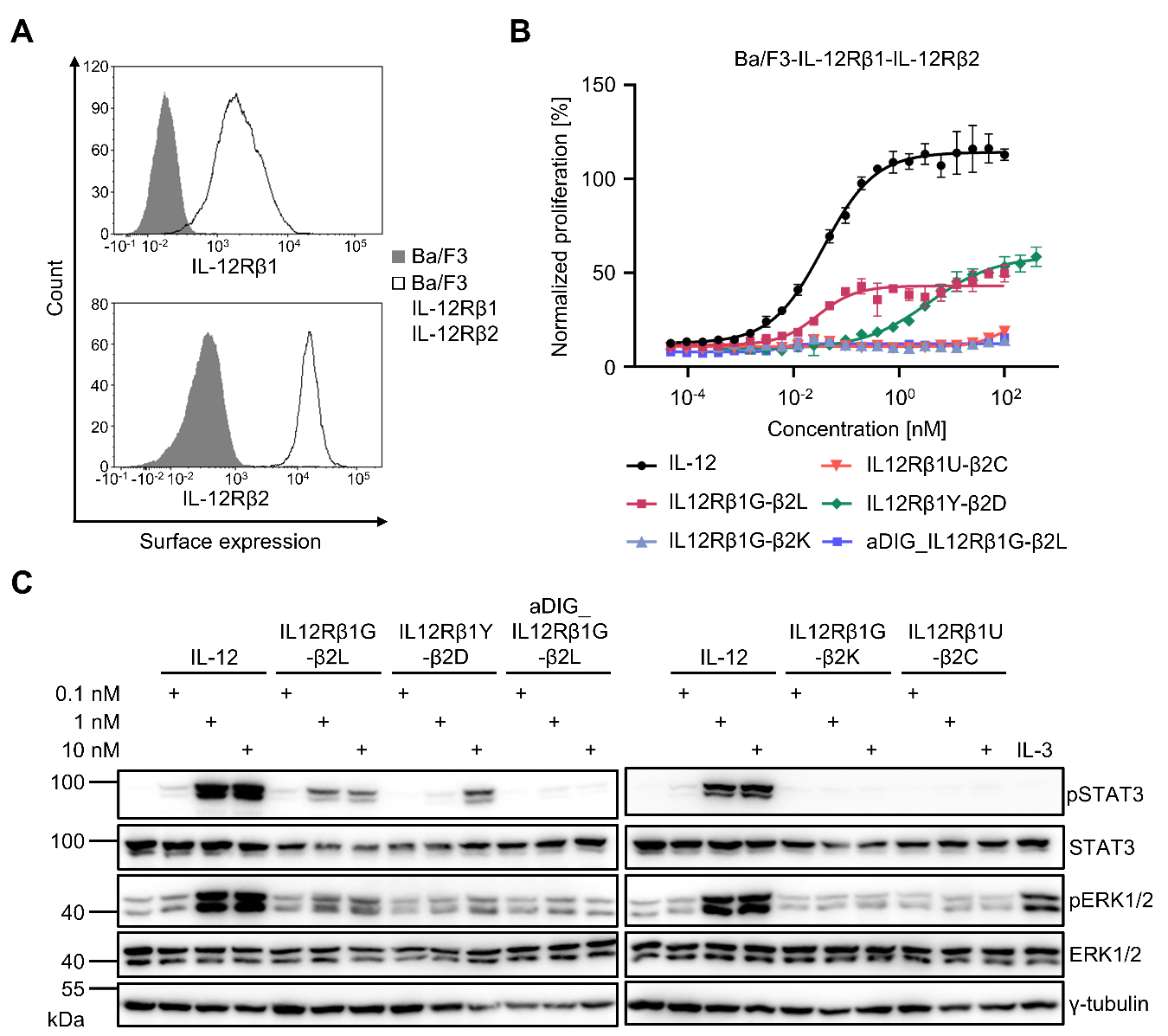


**Figure S10: IL-12 mimetics activate Ba/F3 cells with stable expression of IL-12Rβ1 and IL-12β2.** (A) Flow cytometry analysis of IL-12Rβ1 and IL-12Rβ2 on the surface of stably transduced Ba/F3 cells. The gray-shaded areas indicate Ba/F3 cells (negative control), and the solid lines indicate the respective Ba/F3-IL-12Rβ1-IL-12Rβ2 cell line. (B) Proliferation of Ba/F3-IL-12Rβ1-IL-12Rβ2 cells incubated with increasing concentrations of IL-12 or IL-12 surrogate agonists (IL12Rβ1G-β2L, IL12Rβ1G-β2K, IL12Rβ1U-β2C, IL12Rβ1Y-β2D or aDIG_ IL12Rβ1G-β2L) from 0.00005–100 nM. Cell proliferation was normalized to 0.2 % (v/v) IL-3 induced proliferation. Error bars indicate SD. One representative experiment with four biological replicates out of four independent experiments is shown. (C) STAT3, and ERK activation in Ba/F3 cells expressing IL-12Rβ1 and IL-12Rβ2 treated with 0.1 nM, 1 nM and 10 nM ligand, and 0.2 % (v/v) IL-3 for 30 min. Equal amounts of proteins (50 μg/lane) were analyzed via specific antibodies detecting phospho-STAT3, STAT3, phospho-ERK1/2, ERK1/2 and γ-tubulin. Western blot data shows one representative experiment out of three.

**Table S1: Standard deviations (Emax mean values) and 95 % confident intervals (EC50) of three (NK-92 and NK cells) to four independent experiments/donors.**

|  | NK-92 cells | | | NK cells | | | T cells | | | T_stim_ cells | | | T_stim_ cells | | |
| --- | --- | --- | --- | --- | --- | --- | --- | --- | --- | --- | --- | --- | --- | --- | --- |
| Samples | Mean ± SD absolute pSTAT4+ [%] | EC50 pSTAT4+ [nM] | EC50 95% confident interval | Mean ± SD absolute pSTAT4+ [%] | EC50 pSTAT4+ [nM] | EC50 95% confident interval | Mean ± SD absolute pSTAT4+ [%] | EC50 pSTAT4+ [nM] | EC50 95% confident interval | Mean ± SD absolute pSTAT4+ [%] | EC50 pSTAT4+ [nM] | EC50 95% confident interval | Mean ± SD IFN-γ release [pg/mL] | EC50 IFN-γ release [nM] | EC50 95% confident interval |
| IL-12 | 76.3 ± 4.8 | 0.03 | 0.03 - 0.05 | 20.2 ± 7.0 | 0.03 | n.a. | 4.4 ± 3.4 | 0.002 | n.a. | 45.7 ± 6.4 | 0.003 | 0.002 - 0.006 | 1221 ± 570 | 0.001 | 0.0003 - 0.004 |
| IL12Rβ1A-β2L | 62.2 ± 1.9 | 0.04 | 0.03 - 0.05 |  |  |  |  |  |  |  |  |  |  |  |  |
| IL12Rβ1B-β2E | 15.3 ± 4.8 | n.a. | n.a. |  |  |  |  |  |  |  |  |  |  |  |  |
| IL12Rβ1C-β2B | 55.7 ± 9.0 | 0.08 | 0.04 - 0.15 |  |  |  |  |  |  |  |  |  |  |  |  |
| IL12Rβ1G-β2D | 15.7 ± 8.4 | 0.12 | n.a. |  |  |  |  |  |  |  |  |  |  |  |  |
| IL12Rβ1G-β2G | 49.4 ± 4.5 | 0.06 | 0.04 - 0.08 |  |  |  |  |  |  |  |  |  |  |  |  |
| IL12Rβ1G-β2K | 24.8 ± 8.6 | 0.08 | 0.02 - 0.31 | 0.8 ± 0.4 | 0.06 | 0.008 - 0.312 | 0.2 ± 0.2 | 0.39 | n.a. | 18.0 ± 4.4 | 0.02 | 0.008 - 0.030 | 1034 ± 340 | 0.01 | 0.001 - 0.04 |
| IL12Rβ1G-β2L | 62.1 ± 3.5 | 0.03 | 0.02 - 0.03 | 9.7 ± 5.3 | 0.02 | n.a. | 2.9 ± 1.9 | 0.02 | n.a. | 38.8 ± 6.8 | 0.005 | 0.003 - 0.008 | 1283 ± 495 | 0.003 | 0.0002 - 0.02 |
| IL12Rβ1K-β2C | 47.3 ± 8.5 | 0.01 | 0.004 - 0.018 |  |  |  |  |  |  |  |  |  |  |  |  |
| IL12Rβ1U-β2C | 20.9 ± 7.4 | 1.7 | 0.6 - >100 | 0.6 ± 0.3 | 2.02 | n.a. | 0.3 ± 0.2 | 2.4 | n.a. | 21.3 ± 2.8 | 0.34 | 0.22 - 0.45 | 1110 ± 495 | 0.02 | 0.002 - 0.168 |
| IL12Rβ1Y-β2D | 49.7 ± 6.2 | 10.5 | 8.0 - 27.2 | 5.4 ± 3.4 | 14.8 | > 2.6 | 1.6 ± 0.6 | n.a. | n.a. | 30.1 ± 2.6 | 3.03 | 2.16 - 4.40 | 1037 ± 356 | 0.68 | 0.14 - 3.34 |
| aDIG_IL12Rβ1G-β2L |  |  |  | 3.8 ± 3.9 | 0.11 | > 0.004 | 0.8 ± 0.4 | 0.14 | n.a. | 27.4 ± 2.5 | 0.07 | 0.05 - 0.08 | 1025 ± 346 | 0.01 | 0.001 - 0.078 |
